# Groundwater quality assessment and health risk evaluation for schoolchildren in Mujibnagar, Bangladesh: safe consumption guidelines using artificial neural network modeling

**DOI:** 10.1007/s10653-025-02627-1

**Published:** 2025-07-20

**Authors:** Mohammad Omar Faruk Molla, Md. Anisul Kabir, Md. Kamrul Hossain, Md. Saikoth Jahan, Most. Suria Khatun, Sazal Kumar, Rafiquel Islam

**Affiliations:** 1 Department of Environmental Science and Geography (ESG), Islamic University, Kushtia, 7003 Bangladesh; 2 Department of Applied Chemistry and Chemical Engineering (ACCE), Islamic University, Kushtia, 7003 Bangladesh; 3 School of Environmental and Life Sciences, The University of Newcastle (UoN), Callaghan, NSW 2308 Australia

**Keywords:** Tubewell water, Water quality parameters, Iron and arsenic, Children’s Health Risk, ANN Modelling

## Abstract

Groundwater is a vital source of drinking water in Bangladesh, with tubewells commonly used, particularly in schools. This study assessed the quality of tubewell water in the southwest region, focusing on iron (Fe), arsenic (As), pH, electrical conductivity (EC), and total dissolved solids (TDS). Using artificial neural network (ANN) modeling, daily safe water intake limits for children were estimated based on Fe and As levels. A total of 75 school-based water samples were collected. Fe and As were measured using a Hanna Iron Checker and Hach Arsenic Test Kit. Findings showed that 68% and 48% of samples exceeded WHO and USEPA limits for Fe and As, respectively. In contrast, only 32% and 26.7% of samples surpassed the Bangladesh Water Quality Standards (BDWS) for Fe and As, respectively. Spatial mapping identified Mahajanpur, Bagoan, and Dariapur as hotspots for contamination. TDS showed a strong positive correlation with conductivity (r = 0.92) and a moderate one with salinity (r = 0.7), indicating their interdependence. As and Fe had a weak positive correlation (r = 0.22). PC1 and PC2 explained 56.74% of variance, with Fe and As weakly correlated in PC1 but negatively loaded in PC2, suggesting distinct behaviours influenced by TDS, conductivity, and salinity. Further, Linear regression showed Fe (r = -0.34) and As (r = -0.71) decreased with depth. High health risks were identified for Fe in Mahajanpur and As in Baguan and Dariapur. ANN and Decision Tree Regression models showed 87% accuracy in estimating safe water intake, highlighting Monakhali Union as the most vulnerable area. To avoid health impacts in children, safe consumption levels in Baguan and Monakhali were notably low at 0.59 L/day and 0.39 L/day, respectively. These findings underscore the urgent need for regular monitoring and limiting tubewell water consumption by schoolchildren in the region.

**Supplementary Information:**

The online version contains supplementary material available at 10.1007/s10653-025-02627-1.

## Introduction

Water is essential for sustaining life and supporting human well-being, with safe access to drinking water being a fundamental necessity and a critical component of public health (WHO, 2011; Biswas et al., 2014; Shamsuzzoha et al., 2018). Around a third of the people worldwide depend upon groundwater for drinking and additional household necessities (Nickson et al., 2005). The global water demand is constantly rising, necessitating groundwater utilization (Biswas et al., 2014). Groundwater, specifically tubewell water (TWW), is Bangladesh’s principal drinking water source, fulfilling several needs including household utilities, cooking, and irrigation (Karmoker et al., 2018). However, various factors such as rainfall, surface water quality, geochemical processes, significant chemical concentrations, groundwater recharge, agricultural advancement with disproportionate chemical fertilizer use, and saltwater intrusion in coastal zones negatively affect groundwater quality (Bhavsar & Patel, 2023). Several anthropogenic activities, e.g., industrialization, the overuse of potentially harmful chemicals in agriculture, mining, inefficient land usage, excessive water use, and poor sanitation, lead to this deterioration, ultimately impacting human health (Singh et al., 2021; Onyemesili et al., 2022; Egbueri, 2018, 2020a, 2020b; Egbueri & Enyigwe, 2020; Pal et al., 2022; Mukherjee et al., 2021). Extreme use of nitrogen and phosphorus fertilizer in agrarian fields can degrade the quality of groundwater by the downward movement of chemicals toward the unsaturated zone, which significantly accumulate into plants or crops, and ultimately affect the health of humans (Domagalski & Johnson, 2012; Lord & Anthony, 2002; Schroeder et al., 2004). Other organic pollutants, notably the rising presence of microplastics (MPs), pose a serious threat to groundwater quality. These particles can act as carriers, facilitating the migration of heavy metals, persistent organic compounds, and emerging contaminants into drinking water sources, potentially leading to adverse health effects (Agbasi et al., 2025a).

However, iron (Fe) and arsenic (As) are the two most common trace elements in tubewell water. Iron naturally enters the groundwater through the weathering of rocks and minerals. Some human activities, like landfill leaks, sewage systems, industrial waste, and acid-mine drainage, are responsible too (Thapa et al., 2019). Arsenic (As) is omnipresent in the surroundings, usually in minimal concentrations in all soils, water, air, rocks, and biological tissues (Nriagu & Pacyna, 1988). It is extensively found in the earth’s crust, often occurring as arsenopyrite within iron pyrite-bearing rocks (Ahmed, 2003). Egbueri and Enyigwe (2020) found that the average concentrations of As and Fe were 0.22 mg/L and 0.41 mg/L, respectively, in Southeastern Nigeria. Abba et al. (2024) reported that the average concentration of Fe was found to be 2.32 mg/L, which exceeds the recommended guidelines for drinking water and may pose significant health problems in Al-Hassa Oasis, Saudi Arabia. Another study conducted by Agbasi et al. (2024) in Southeastern Nigeria reported that 53.6% of groundwater samples surpassed the recommended values of Fe. Further, Aswal et al. (2023) and Aswal et al. (2024) reported that the average concentrations of As were 0.18 µg/L and 3.18 µg/L in the impacted area of India.

In Manikganj district, Bangladesh, Tasneem et al. (2020) reported that considerable Fe contamination was observed in the Singair Upazila’s groundwater (0.175 – 13.865 mg/L). Ghosh et al. (2020) also found that Fe concentration varied (0.02 – 6.2 mg/L) across different locations in the Jashore district. Hossain and Sivakumar (2006) documented that the arsenic levels varied with a range of 1 to 224 ppb in several locations across Bangladesh at different depths (23–45 m). 12.6% of the tested samples collected from the tubewell within 13,423 Bangladeshi houses exceeded the established arsenic limit of drinking water (DPHE, 2009). Hossain et al. (2023) found that excess Fe concentrations were present in Kushtia Sadar’s 77% and 80% of the collected groundwater samples, respectively, above the standard levels by BDWS and WHO.

Schoolgoers are vulnerable due to As and Fe consumption through drinking water is a high matter of concern, causing their respiratory, digestive, reproductive, central nervous, and immune systems to be more sensitive than adults (Burtscher & Schuepp, 2012; Salvi, 2007; Madureira et al., 2016). Chronic exposure to As causes an impact on children’s Intelligence Quotient (IQ), damage to parenchymal cells, endocrine disruption, and cardiovascular systems (Gupta et al., 2020; Rahman et al., 2009; Xu et al., 2021). Prolonged exposure to high iron and manganese levels can lead to several health problems for adults and children, e.g., Parkinson’s disorder, Cardiovascular disease, Huntington’s disorder, Hyperkeratosis, Alzheimer’s disease, Pigmentation changes, Diabetes mellitus, Respiratory issues, Kidney and liver disorders, and Neurological disease (Ghosh et al., 2020; Goldhaber, 2003; Torres-Agustín et al., 2013; Wasserman et al., 2006). The hazard quotient (HQ) values indicate potential non-carcinogenic health risks associated with Fe intake, ranging from 0.16 to 0.92 for adults and 0.39 to 2.13 for children in Kushtia Sadar (Hossain et al., 2023). Similarly, Islam et al. (2023) reported that As contaminants are found in almost 63% of the Gangni Union, and the value of HQ ranges between 0.0277 and 6.033 for children, which was higher than for adults (0.0092–2.011). Additionally, several studies have shown that in Bangladesh’s coastal regions, primary schools’ drinking water is contaminated with As, Fe, and Cl^−^ (ion)  (Rahman & Hashem, 2019a, 2019b), resulting in long-term risks of cancer and non-cancer disorders for school-age children.

Meherpur was enrolled as the ‘worst-affected’ district in Bangladesh, accounting for 60% of the area afflicted by As with an average concentration of 116 μg/L (BGS, 2001). As a developing country, Bangladesh’s government schools use tubewells as their prime drinking water source without further purification. A little study has been conducted regarding the groundwater’s As contamination in the Meherpur district, such as Haque et al. (2019) tested As concentration among 14 tubewells where the average depth is 38.75 m in Amjhupi Union, Meherpur. Another study was conducted by Rahman et al. (2023), considering 10 tubewell samples where the average depth was 59.7 m. However, no specific research was revealed on the groundwater quality of the school’s tubewell and evaluating health risks of schoolgoers, particularly focusing on Fe and As contamination. Additionally, model-based evaluations to determine safe water consumption limits for school children are lacking. To fill the research gap, this study aims to evaluate the status of groundwater quality and its associated potential health risks for schoolgoers from Fe and As exposure. Also, the estimations of threshold consumption or the safe daily water intake for school children to avoid health impacts using artificial neural network (ANN) modeling are entirely unknown. This study’s findings will offer crucial insights for environmental and health policymakers in assessing drinking water quality, evaluating health risks posed by toxic substances to school children, even in adults, and determining safe consumption limits, purification strategies, or sustainable alternatives to mitigate long-term health effects for daily consumers.

## Materials and methods

### Study area and field sample collection

**Fig. 1:**
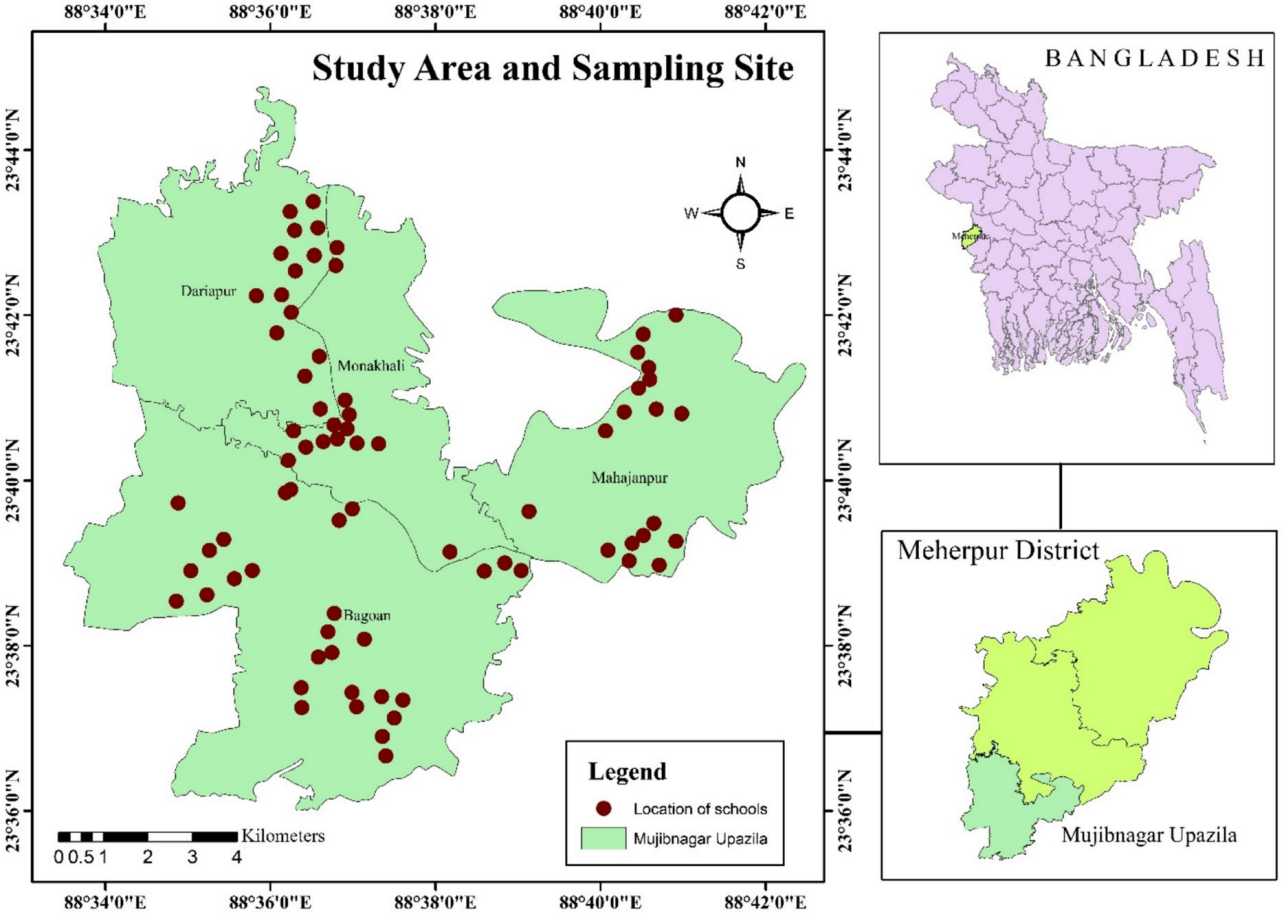
Study area and distribution of sampling points (n = 75) in the south–western part of Bangladesh

Mujibnagar Upazila has been selected for this study, located in the Meherpur district of Bangladesh, between 23°36′N and 23°45′N, and between 88°34′E and 88°43′E. The total area and population of the study region are approximately 112.68 square kilometers and 99143, respectively (BNP, 2024). It comprises four unions (Bagoan, Dariapur, Mahajanpur, and Monakhali), including Sarashati Canal, Datpur Beel, and Bhairab River as the study area’s surface water sources (Banglapedia, 2023). Despite being almost flat, the terrain has a gentle southward slope from the north, and thus, surface geology and soil structure significantly influence the shallow aquifers, controlling the pathways and timing of groundwater recharge (WARPO, 2000; MPO, 1987). The geology of this area is primarily alluvial, characterized by gray and brown sand, silt, and clay (Haque et al., 2017). Since the average humidity is relatively high, wet weather is notable for most of the year, and evapotranspiration is comparatively low. Following purposive sampling, 75 primary schools were selected to collect the water samples from the tubewell. After physical observation at each selected point, 250 mL of each water sample was taken in amber glass bottles (replicates, n = 3) from the school’s TWW. Before using these amber bottles, all of them were washed thoroughly with disinfectant (2% Citranox solution), soaked in 5% hydrochloric acid overnight, and rinsed with deionized water to ensure no contaminants were available. The amber glass bottles were securely sealed with strong surgical tape to restrict further sample contamination during collection. The collected water samples were immediately stored in the icebox containing Eskys to maintain their integrity and moved to the Environmental Analysis Laboratory within 4–5 h of collection. Additionally, the Global Positioning System (GPS) devices were utilized to collect the precise geolocation of the sites for sampling. The present study area and locations for sampling are depicted in Fig. 1.

### Laboratory analysis

This study used a digital Hanna HI-721 (Hanna Instrument: Iron Colorimeter—Checker® HC India) Iron Checker handheld colorimeter, ranging from 0.00 to 5.00 mg/L, and the Hanna HI-721 reagents to analyse the iron concentrations, consistent with the research by Hossain et al. (2023). Arsenic concentration is assessed employing the Hach EZ Dual-Range Arsenic Test Kit (Hach- Product Number: 100735), with a 0.00–0.5 mg/L detection limit, followed by Islam et al. (2023). Standard safety procedures and guidelines were also followed to examine the sample. The additional water quality parameters, such as pH, Total Dissolved Solids (TDS), and Electrical Conductivity (EC), were measured in situ at each sampling location using a portable multi-parameter water quality device, presented in Table S1.

### Statistical analysis

All statistical tests were computed and prepared using Origin Pro (Statistical Software-V24). For this study, Pearson’s correlation was utilized to identify the relationship among the groundwater quality parameters. The correlation coefficient varies between − 1 and + 1, where + 1 refers to the ideal relationship, and − 1 signifies a negative correlation among variables (Abounaima et al., 2020; Ratner, 2009). Principal Component Analysis (PCA) is a prevalent multivariate statistical method that transforms log_10_-scaled indicator variables into independent principal components, facilitating the identification of significant variables and dimensionality reduction while preserving most of the original information (Arslan, 2013; Shrestha & Kazama, 2007). This study used PCA to observe the relationship and variability among the water quality parameters throughout the study area’s various unions. Linear Regression analysis is a statistical technique used to solve environmental problems and identify the relationship between two variables (Kumari & Yadav, 2018). Linear regression was employed in this study to ascertain the Fe and As concentration variations with depth of the tubewell.

### Geospatial analysis

This study used graduated symbols, which vary in size, to demonstrate a quantitative variation between mapped features (Brewer, 2006). The data was classified into ranges, and then a symbol size was assigned to each range to represent it. Symbol size is an effective tool to show differences in a phenomenon’s magnitude because naturally larger symbols are related to a greater amount of something (ESRI, 2024). In this study, graduate symbols were implemented to represent and visualize the concentrations of Fe and As at every primary school in the study area using ArcGIS 10.8 to observe, and this method was employed as it suitably indicates the magnitude of the contaminants at every school location.

### Non-carcinogenic health risk analysis

The US EPA’s recommended method was utilized to calculate the non-carcinogenic risks to health for boys and girls applying the metals’ chronic daily intake (CDI) and the hazard quotient (HQ) (US EPA, 2004, 2011). Based on Eq. 1, the CDI is computed, which is shown below (US EPA, 2011):

1 $${\text{CDI}} = \frac{{\left( {{\text{C }} \times {\text{ IRwater }} \times {\text{ EF }} \times {\text{ ED}}} \right)}}{{\left( {{\text{BW }} \times {\text{ AT}}} \right)}}$$

where, C = The Arsenic and Iron concentration (mg/L) in each primary school’s drinking water; IR _water_ = The water ingestion rate (1.0 L per day (d)^−1^) for each primary school’s children; EF = The exposure frequency (230 d/year); ED = The exposure duration (5 years) AT = The average time (1,150 days); BW = The body weights for school children (6–10 years old) where the mean body weights of girls and boys are approximately 20 kg and 24.68 kg, respectively (NCHS, 2001).

According to Hossain et al. (2013), to estimate health risks during school time, 1.0 L of water intake was considered in this study, although children typically intake 2.1 L of water. Based on annual school opening days, the exposure duration was calculated. The HQ is calculated for this study using Eqs. 2 and 3 as follows (US EPA, 2004):

2 $${\text{HQ}} = \frac{{{\text{CDI}}}}{RfDo}$$

3 $${\text{HI}} = { }\sum {\text{HQ}} = {\text{ HQAs}} + {\text{HQFe}}$$

where, R_f_D_o_ = The oral reference dosage (mg/kg/d), and the R_f_D_o_ value for iron and arsenic were 0.7 mg/kg/d and 0.0003 mg/kg/d, respectively (US EPA, 2020). If the HQ value is less than 1, the exposed populations are considered safe from the trace metal (loid)’s specific harmful effects; and HQ > 1 might have detrimental health impacts on the people who are exposed. However, a cumulative measure that considers the aggregate effects of individual metals’ HQs is the hazard index (HI). HI > 1 denotes the likelihood of a non-carcinogenic risk to health, whereas HI ≤ 1 suggests no probable risk to health (US EPA, 2001).

### Establishment of numerical models and methods validation

Machine learning is developed as a reliable method for understanding and analyzing a dataset’s behavior, utilizing the data’s interrelated nature and comprehending how this will respond to the alterations of the independent factors (Akash et al., 2024). It may imitate a unique circumstance to predict and understand any occurrence in the future. Artificial neural networks and linear regression were used to identify the data’s concealed structure and offered complex clustering of data and building neural networks (Shi & Shen, 2022). This section includes different regression methods that assume the result is a straight combination of the features, as shown in Eq. 4 (Nguyen et al., 2018).

4 $$y\left( {w, x} \right) = w0 + w1x1 + w2x2 \ldots \ldots + wpxp$$

where y is the safe consumption limit or output of the model, x is the vector of input features, w0 is the intercept (bias term), w (w1, w2………, wp) is the vector throughout the module assigned as intercept and coefficient, and p is the number of input features. Linear regressions generate linear models, including coefficients, that aim to minimize the sum of squared differences between the dataset’s actual and predicted target values using the linear approach.

On the contrary, ANN uses the following formula (Eq. 5) to calculate the loss function’s gradient (Akash et al., 2024):

5 $$\omega = \omega - \eta \left[ {a\left\{ {{{\delta R\left( \omega \right)} \mathord{\left/ {\vphantom {{\delta R\left( \omega \right)} {\delta \left( \omega \right)}}} \right. \kern-0pt} {\delta \left( \omega \right)}}} \right\} + \left\{ {{{\delta Loss} \mathord{\left/ {\vphantom {{\delta Loss} {\delta \omega }}} \right. \kern-0pt} {\delta \omega }}} \right\}} \right]$$

where, $\omega$ is the vector of weights in the neural network, and η is the learning rate that limits the size of the step within that parameter space search. $a$ – the scaling factor or regularization strength, R ($\omega$) is a regularization function, Loss = The function of loss employed for that network. $\delta /\delta \omega$– the gradient operator, pointing us in the direction of steepest descent, where progress lies.

The neural network is more capable of handling numerical data, and using encoded numerical instead of categorical data improves the model’s performance (Akash et al., 2024). So, instead of textual categorical data, the data was encoded with a representative number. The required data for decoding the feed is provided in Table S5. Safe water for drinking may be affected by several heavy metal concentrations, and their concentration in groundwater is deeply dependent on the geographical location (Nouri et al., 2008). A neural model can effectively analyze the existing relationship among the provided data to simulate the possible content of heavy metal in the radius and determine the concentration at which it may create long-term and irreversible effects on children. Jupyter Notebook 7.0.8 was used by Anaconda distribution for utilizing machine learning models library numpy 2.2, pandas 2.2.3, sci-kit learn 1.6, and pytorch 2.6.0 for execution of the numerical model.

The textual data, including contaminants’ names, types, etc., were encoded into the numerical data categories that fed the data to the model. In addition, a flag variable was created for each metal (loid)s containing binary values, e.g., 0 or 1. “0” implies the concentration is below the acceptable limit. Conversely, “1” indicates surpassing the acceptable limit. Necessary information is included in the supplementary data to decode textual data (Table S5). For validation, 90% and 10% of the data were split into training and testing sets, respectively. For the model’s validity, 10% of the testing dataset was utilized after feeding the model with 90% of the training dataset. The Shapley (SHAP) value has been used for the model’s interpretation, which allows decoding of the numerical outputs and their driving forces. Consequently, this may have a minimal impact on data collection and presentation; finally, every sampling point has distinct safe consumption limitations that need to be considered cautiously. The computer modeling-based technique, along with potential guidelines, has been analyzed based on the implemented model; additionally, it utilizes probable consumption per day according to the status of Fe and As contamination of the distinct water samples and recognizes no risks to human health. A potential safe intake threshold can be assessed with the following Eq. 6, assuming the THQ = 1 (Akash et al., 2024).

6 $$FIR = \left[ {{{\left( {BW \times Rf_{Do} \times AT} \right)} \mathord{\left/ {\vphantom {{\left( {BW \times Rf_{Do} \times AT} \right)} {\left( {EF \times ED \times C} \right)}}} \right. \kern-0pt} {\left( {EF \times ED \times C} \right)}}} \right] \times 10^{3}$$

where, FIR = the water consumption rate (L/day); EF = The exposure frequency (230 d/year); ED = exposure duration (5 years); C = The arsenic and iron concentration in water (mg/L); BW = average body weight; AT = the average non-carcinogen time (230 d/year × number of the exposure years).

Figure S1 shows a simple diagram of the ANN processes and how data is grouped using the stimulated As and Fe concentration data, while considering the THQ values that highlight the data’s hidden features.

The dataset was partitioned into a training set (90%) and a testing set (10%) to evaluate predictive performance. The model was trained on the training set and evaluated on the unseen testing data, simulating an effective model. This simple validation method effectively avoids overfitting when the data distribution is representative of the real population (Kuhn & Johnson, 2013). In addition to the train-test split, tenfold cross-validation was conducted. The dataset was divided into 10 equal subsets, and the model was iteratively trained on 9 subsets while tested on the remaining one. The process was repeated 10 times, ensuring that every sample was used for validation once. The average accuracy across all folds was recorded, minimizing variance due to random data splits, and offers for all parts were noted, reducing differences caused by random data divisions and providing a more reliable measure.

## Results

### Groundwater parameters

The results of the groundwater quality parameters of the study area are presented in Table 1. The pH range was 6.12 to 7.8 with an average of 6.85 ± 0.24, and all the samples followed the acceptable ranges of WHO (2017) and US EPA (2009). The EC showed values between 2.72 and 1767 µS/cm; the average value of EC was 900.27 ± 290.48 µS/cm, which surpassed the WHO standard threshold of 250 µS/cm significantly in 97.3% of samples, exhibiting elevated levels of dissolved ions. The measured TDS levels varied from 1.37 to 882 mg/L, averaging 460.50 ± 144.30 mg/L, where 36% and 88% of samples exceeded the standard thresholds of US EPA (500 mg/L) and WHO (600–1,000 mg/L), respectively.

**Table 1:** Descriptive statistics of measured water quality parameters of the study area

This study			Water quality standard			Number of samples exceeding water quality standard			Percentages of samples exceeding water quality standards		
Parameters (Unit)	Range	Average ± SD	WHO *(a)*	US EPA *(b)*	BDWS *(c)*	WHO *(a)*	US EPA *(b)*	BDWS *(c)*	WHO *(a)*	US EPA *(b)*	BDWS *(c)*
pH	6.12–7.8	6.85 ± 0.24	6.5–8.5	6.5–8.5	6.5–8.5	0	0	0	0	0	0
EC (µs/cm)	2.72–1767	900.27 ± 290.48	250	–	–	73	–	–	97.3	–	–
TDS (mg/l)	1.37–882	460.50 ± 144.30	600–1000	500	1000	66	27	–	88	36	–
Fe (mg/l)	0–3.66	0.94 ± 0.92	0.3	0.3	0.3–1.0	51	51	24	68	68	32
As (mg/l)	0–0.175	0.03 ± 0.05	0.01	0.01	0.05	36	36	20	48	48	26.7
Depth (feet)	100–560	311.20 ± 126.14	–	–	–	–	–	–	–	–	–

EC: Electrical Conductivity; TDS: Total Dissolved Solids; Fe: Iron; As: Arsenic; S.D: Standard Deviation; WHO: World Health Organization; USEPA: United States Environmental Protection Agency; BDWS: Bangladesh Water Quality Standard; ECR: Environmental Conservation Rule *(a) *WHO, 2017, (b) US EPA, 2009, and (c) BDWS, 1997 (ECR)

The iron concentration ranged between 0 and 3.66 mg/L, with a mean value of 0.94 ± 0.92 mg/L. About 68% of the tested samples surpassed the WHO (2017) and US EPA (2009) recommended range of 0.3 mg/L, and 32% exceeded the BDWS standard range of 0.3–1.0 mg/L. On the Contrary, arsenic concentration levels ranged between 0 and 0.175 mg/L, including a mean value of 0.03 ± 0.05 mg/L. Of the samples, 48% were above the WHO and US EPA standards of 0.01 mg/L, whereas 26.7% surpassed the 0.05 mg/L BDWS threshold, highlighting significant contamination concerns. Lastly, the groundwater samples’ depth varied from 100 to 560 feet, averaging 311.20 ± 126.14 feet. Detailed information on the collected and analyzed groundwater samples is presented in Table S2.

### Spatial distribution of Fe and As concentrations

**Fig. 2:**
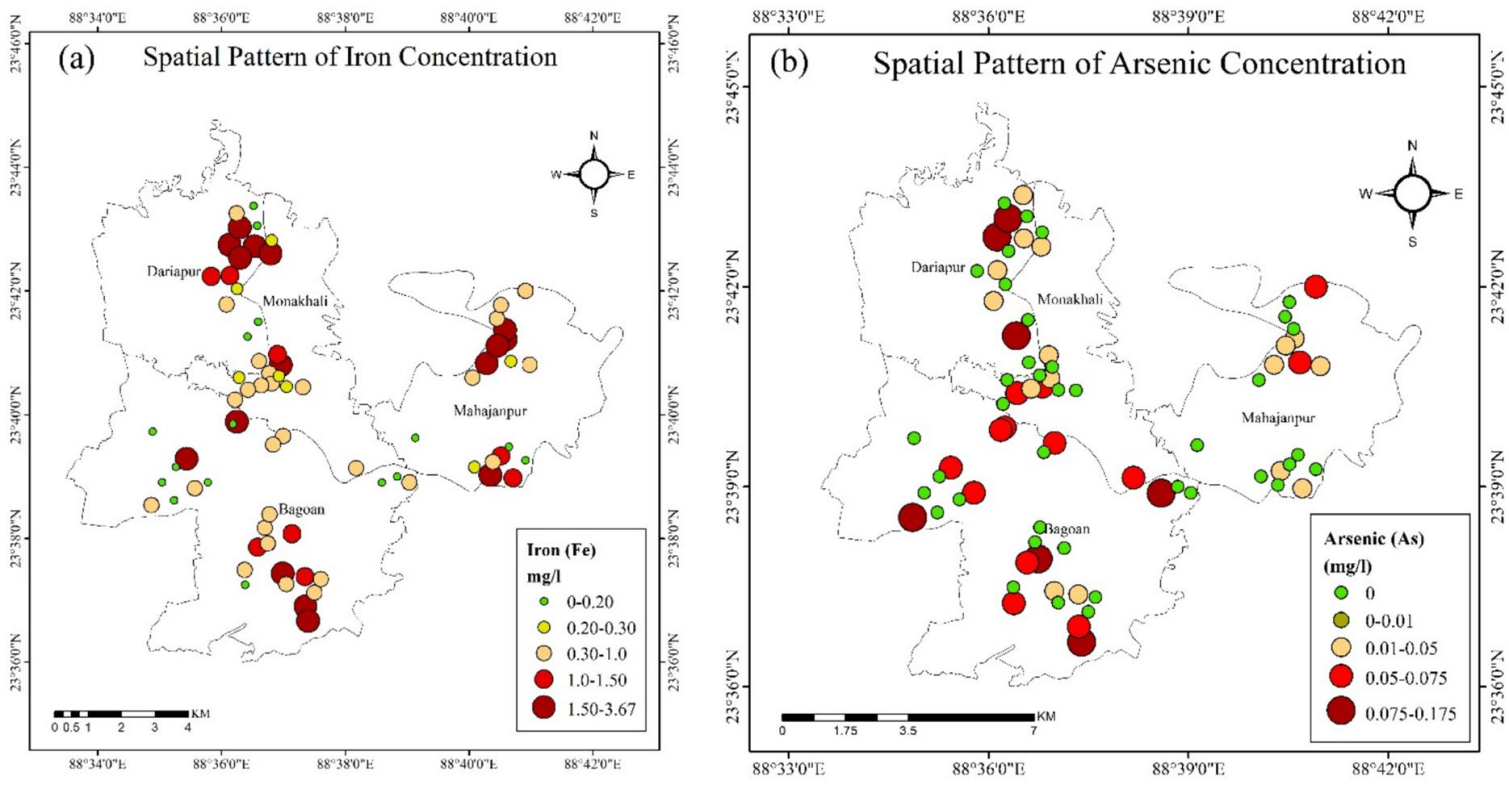
Spatial distribution pattern and variation of (**a)** iron and (**b) **arsenic concentration of the study area

The spatial distributions of iron and arsenic concentrations across the study area are presented in Fig. 2. Figure 2a, which depicts iron concentrations, clusters the highest concentrations (1.50–3.67 mg/L) in several regions, particularly around Monakhali, Dariapur, and Bagoan, with a dark red circle indicating elevated levels. Yellow, orange, and red circles mark the widely dispersed moderate iron concentrations (0.2–1.50 mg/L), while green circles depict the sparse and predominantly isolated areas of the lowest concentrations (0.0–0.20 mg/L).

In Fig. 2b, representing arsenic concentrations, the spatial pattern follows a similar trend, with high arsenic concentrations (0.075–0.175 mg/L) marked by dark red circles in areas such as Bagoan and Dariapur. Moderate arsenic levels (0.01–0.075 mg/L) are more spread across the study area, as shown by the orange and red circles. On the contrary, the lowest concentrations, e.g., 0–0.01 mg/L, are shown in green and tend to be located in relatively isolated zones.

### Correlation among groundwater quality parameters

**Fig. 3:**
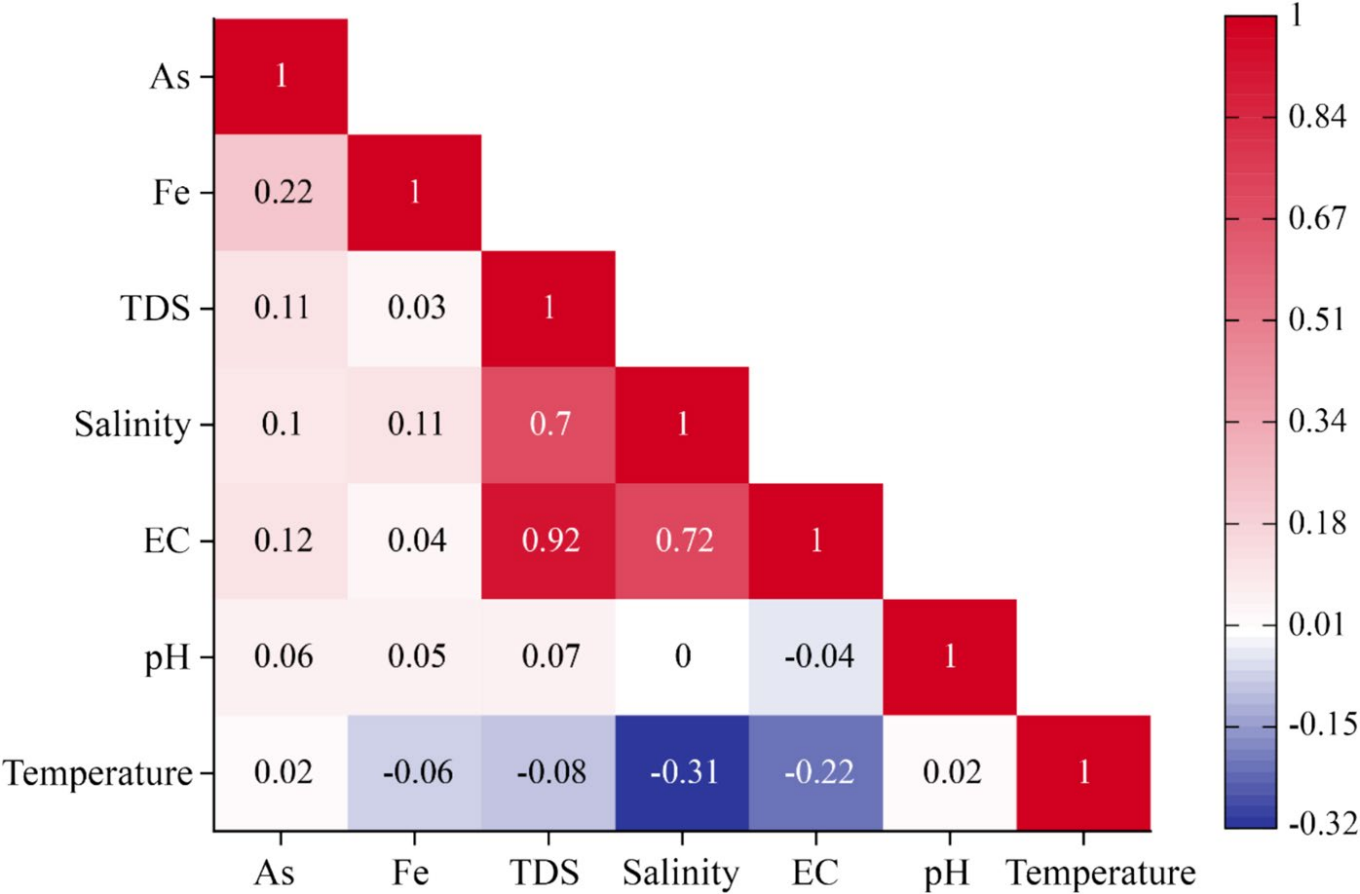
Pearson correlation analysis among water quality parameters

Pearson correlation coefficients between various groundwater quality parameters, including arsenic (As), iron (Fe), total dissolved solids (TDS), temperature, salinity, pH, and electrical conductivity (EC), utilizing 75 samples presented in Fig. 3. A strong positive relationship was observed between TDS and electrical conductivity (r = 0.92), and a moderate correlation was found between TDS and salinity (r = 0.7), highlighting that higher TDS levels in groundwater are related to increased salinity and conductivity. Conversely, As and Fe show a weak positive correlation (r = 0.22), implying a metal individuality and a small association between Fe and As concentrations. Relationships between temperature and other parameters display weak correlations, both positive and negative, suggesting minimal direct influence. These correlations provide significant insights into the interconnectivity of groundwater quality parameters and may inform targeted strategies for managing and monitoring groundwater resources, particularly concerning As and Fe contamination.

### Principal component analysis (PCA)

**Fig. 4:**
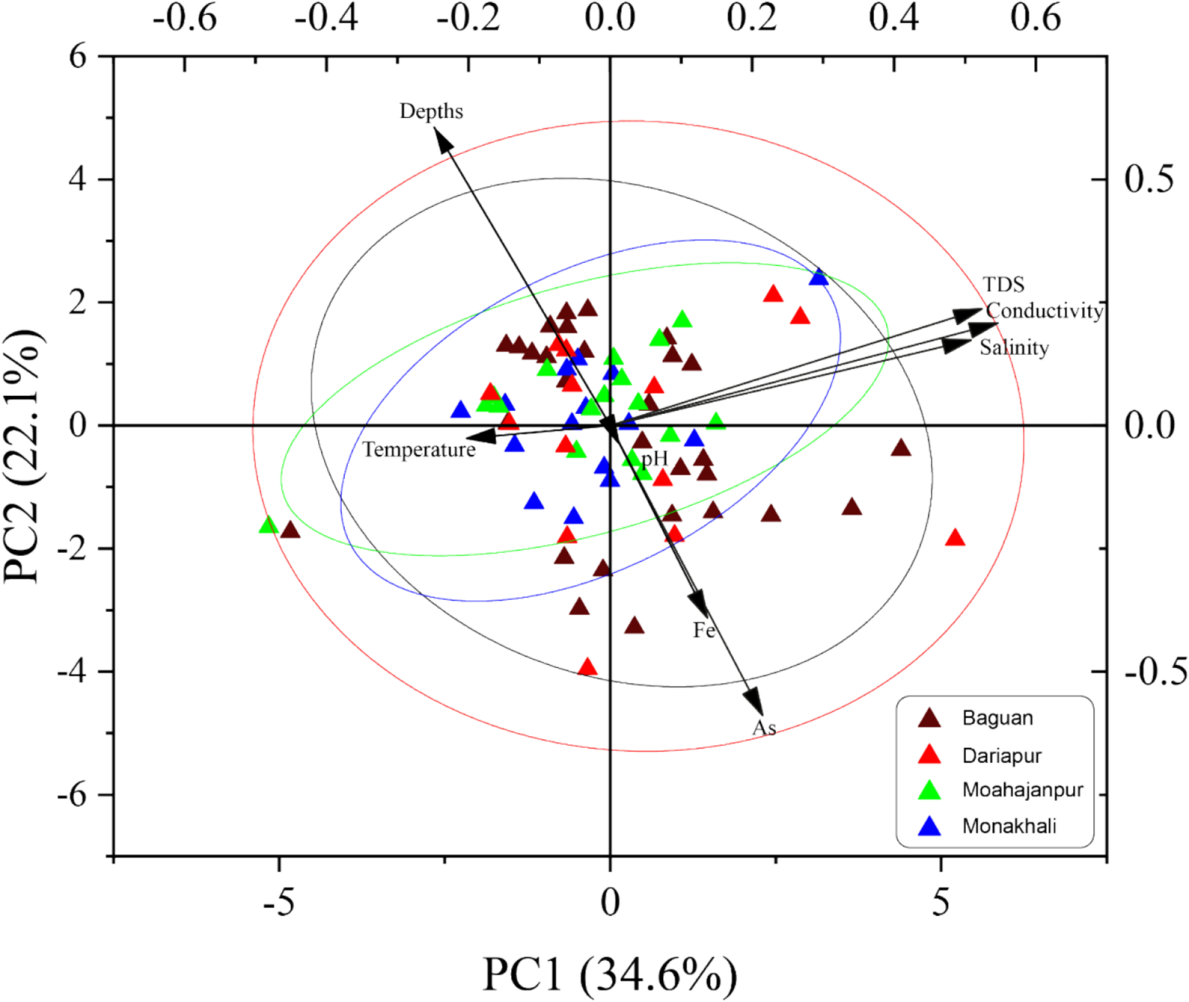
Principal component analysis using water quality parameters across different unions of the study area

A biplot generated from a Principal Component Analysis (PCA) of groundwater quality data from four distinct unions (Bagoan, Dariapur, Mohajanpur, and Monakhali) is illustrated in Fig. 4, emphasizing the interconnections among diverse water quality indicators. The PCA identified principal components (PCs), PC1 and PC2 accounted for the majority of the variability, explaining 34.61% and 22.13% respectively, summing to 56.74% of the total variance. This indicates that more than half of the variation in water quality across sites can be interpreted using these two components, suggesting a high degree of dimensional reduction and interpretability ( Table S3). PC1 loading moderately correlates with total dissolved solids (TDS), salinity, and conductivity, with loads of 0.524, 0.508, and 0.5464, respectively. However, a moderate load was observed in PC2, within the same cluster of those parameters, indicating that these metrics originate from a shared source or are influenced by analogous geological processes. Simultaneously, PC2 is characterized by metrics like temperature, pH, and depth, which signify their independent contribution to the overall variability in the dataset, distinct from the factors represented by PC1. Fe and As exhibit a weak correlation in PC1, with positive loads of 0.214 and 0.136, however, in PC2, these are − 0.589 and − 0.391 negative loads, within the same cluster, suggesting these metals individuality, and may be affected by analogous processes with TDS, EC, and salinity, but to a lesser degree. Bagoan, Mohajanpur, and Monakhali have distinct and consistent clusters, except the Dariapur union, in which parameters are randomly distributed.

### Linear regression of iron and arsenic concentration with depth

**Fig. 5:**
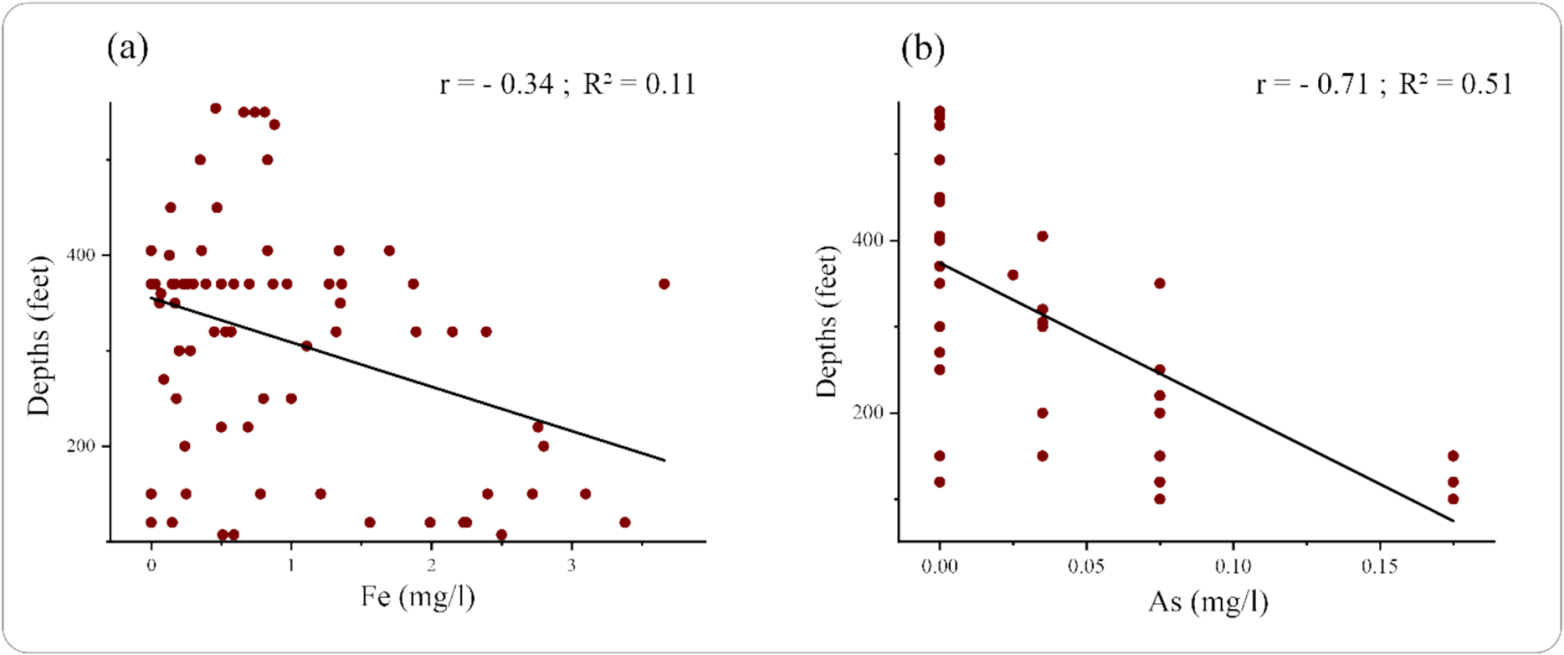
Variation of (**a)** iron and (**b)** arsenic concentration with depth of the tubewell

The variations of iron and arsenic concentrations with the depths of the tubewell as illustrated in Fig. 5. Figure 5a shows a moderate negative correlation (R^2^ = 0.11; r = − 0.34) between Fe and depths, suggesting that increasing the tubewell’s depth will decrease iron concentrations. In contrast, Fig. 5b indicates a significant negative relationship with a linear trend between depths and the concentration of arsenic (R^2^ = 0.51; r = − 0.71). This also suggests that an increase in the tubewell’s depth will decrease arsenic concentrations, indicating that the geological layer of the groundwater makes shallower water more vulnerable.

### Hazard quotients (HQ) based on health risks

**Fig. 6:**
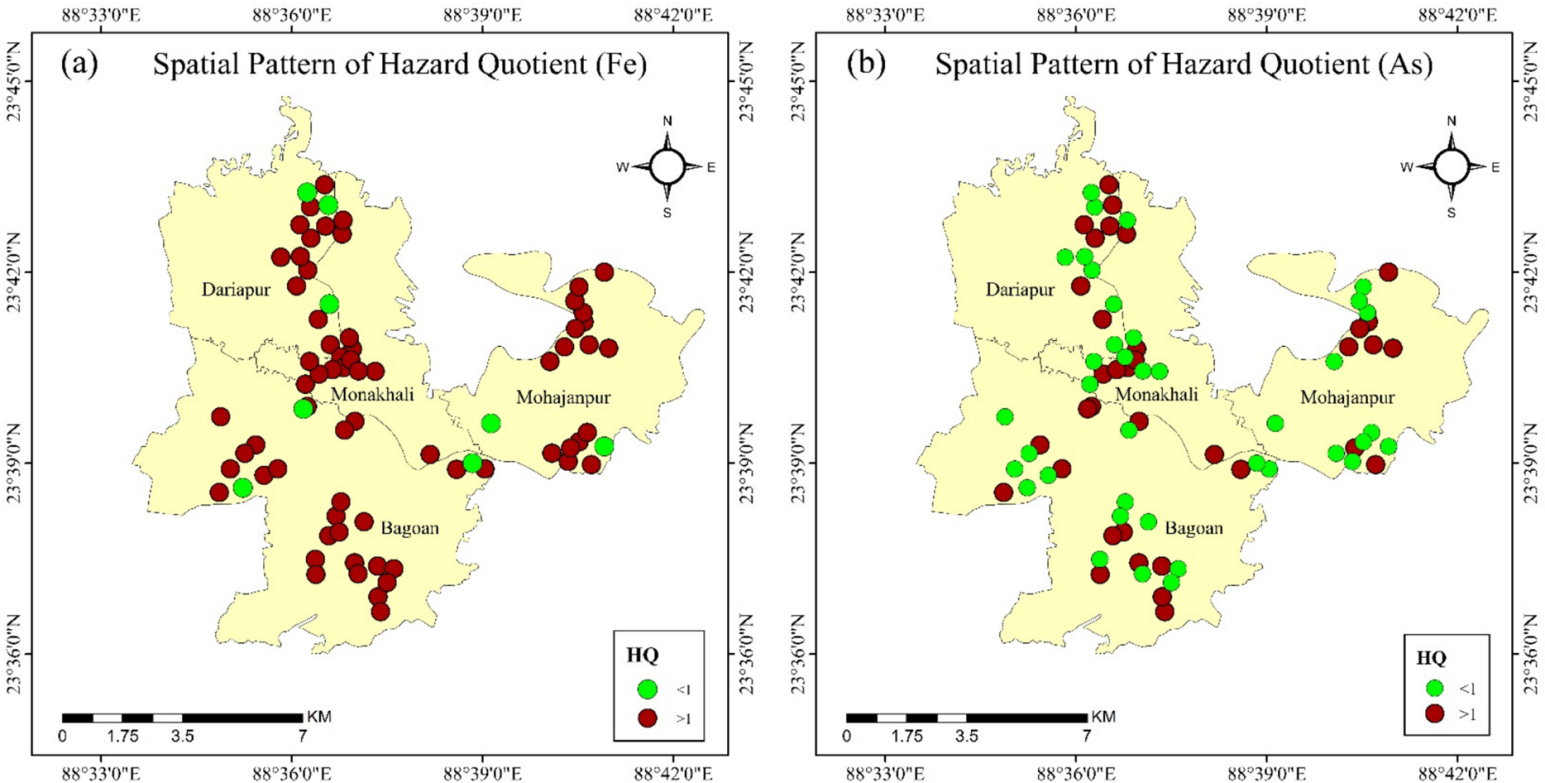
Spatial pattern of hazard quotient due to the exposure of (**a)** iron (**b)** arsenic

The spatial patterns of HQ for iron and arsenic for each sampling point throughout the study region are depicted in Fig. 6. The spatial pattern of Fe hazard quotients in Fig. 6a, where red circles indicate HQ values greater than 1, represents areas with potential health risks because of high iron concentrations. Most sites display red circles with some clustered areas, signifying widespread elevated iron levels. In contrast, only a few green circles denote HQ values less than 1 in some scattered locations. Figure 6b exhibits the spatial distribution of arsenic hazard quotients, with HQ values larger than 1 depicted by red circles. However, notable clusters of increased arsenic risk are found in some specific areas.

The HQ and HI, based on the union, revealed the entire study area’s significant potential health risks. 26 out of 29 samples in Bagoan displayed an HQ > 1 for iron (Fe), with concentrations ranging between 1.24 and 26.29, while 14 samples also surpassed HQ > 1 for arsenic (As), with levels varying from 7.78 to 38.89 (Table 2). Similarly, Monakhali exhibited 15 samples of iron (2.19–25.9) with HQ > 1, and 7 samples of arsenic (7.78–16.67) with HQ > 1. In Moahajanpur, 15 samples of iron (1.62–34.86 mg/L) showed an HQ > 1, and 8 samples of arsenic (7.78–16.67) exceeded HQ > 1. 11 out of 14 samples of iron (1.43–32.19) indicated an HQ > 1 in Dariapur, while 7 samples of arsenic (5.56–38.89) had HQ > 1. The study areas’ Hazard Indices (HI) further emphasized the overall risks posed by these contaminants, with the highest observed HI values in Bagoan (0.57–60.13), Monakhali (2.19– 30.54), Mahajanpur (0 – 34.86), and Dariapur (0–71.08). Moreover, the Hazard indices (HI) for each sampling point are depicted in Table S4. These results indicate the prevalent risks of groundwater contamination, with iron and arsenic concentrations exceeding acceptable safety criteria in numerous samples, particularly in the Bagoan and Dariapur unions.

**Table 2:** Variations of iron and arsenic concentration in Tubewell waters in the tested sites in Bangladesh and worldwide

GW quality	Sampling sites	Fe (mg/L)	As (mg/L)	References
National	Mujibnagar (n = 75)	0.94 (0–3.66)	0.03 (0–0.175)	*This study
	Dinajpur (n = 13)	1.7 (0.05–5.6)	0.014 (0.002–0.054)	Rana et al. (2022)
	Gaibandha (n = 7)	2.22 (0.89–7.4)	0.025 (0.012–0.039)	Rana et al. (2022)
	Kurigram (n = 9)	3.12 (0.16–8.8)	0.093 (0.017–0.176)	Rana et al. (2022)
	Lalmonirhat (n = 5)	1.62 (0.98–2.6)	0.007 (0.005–0.01)	Rana et al. (2022)
	Nilphamari (n = 6)	3.45 (1.5–6.8)	0.014 (0.004–0.08)	Rana et al. (2022)
	Panchagar (n = 7)	5.08 (2.3–10)	0.011 (0.003–0.054)	Rana et al. (2022)
	Rangpur (n = 8)	3.98 (1.3–9.1)	0.146 (0.005–0.994)	Rana et al. (2022)
	Thakurgaon (n = 5)	2.61 (0.63–4.73)	0.009 (0.002–0.014)	Rana et al. (2022)
	Sylhet (n = 38)	5.91 (0.01–15.95)	0.021 (0.00012–0.07)	Ahmed et al. (2019)
	Kushtia (n = 32)	0.25 (0.04–1.45)	0.017 (0.001–0.098)	Rahman and Rahaman, (2018)
	Magura (n = 20)	2.15 (0.09–8.44)	0.016 (0.001–0.055)	Rahman et al. (2016)
	Narail (n = 49)	2.07 (0.011–8.67)	0.091 (0.001–0.321)	Mohana et al. (2020)
	Khulna (n = 35)	2.18 (0.17–8.58)	0.002 (0.0004–0.0035)	Rahman et al., (2021a, 2021b)
	Satkhira (n = 60)	4.2 (0.03–13.42)	0.109 (0.001–0.25)	Rahman et al. (2021a)
Global	Australia	Up to 1300	0.05 – 0.34	Adeloju et al. (2021) Appleyard et al. (2004)
	Ghana	0.00 –13.7	< 0.001 – 0.18	Acquah et al. (2025), Herath et al. (2016)
	Finland	< 0.03 – 29.90	0.017 – 0.98	Luoma et al. (2024) Shankar et al. (2014)
	India	0.10 – 9.04	0.18 – 3.19	Sharma et al. (2021) Aswal et al. (2023)
	Nepal	< 0.10 –7.22	Up to 2.62	Sarkar et al. (2022) Shankar et al. (2014)
	USA	0.15 – 25.0	Up to 2.6	Johnson et al. (2018) Adeloju et al. (2021)
	Italy	0.00075 – 3.39	0.0001– 6.94	Triassi et al. (2023) Adeloju et al. (2021)
	China	0.03 – 6.80	0.05 – 4.44	Guo et al. (2014) Adeloju et al. (2021)

### Computational modelling-based drinking water recommendations

**Fig. 7:**
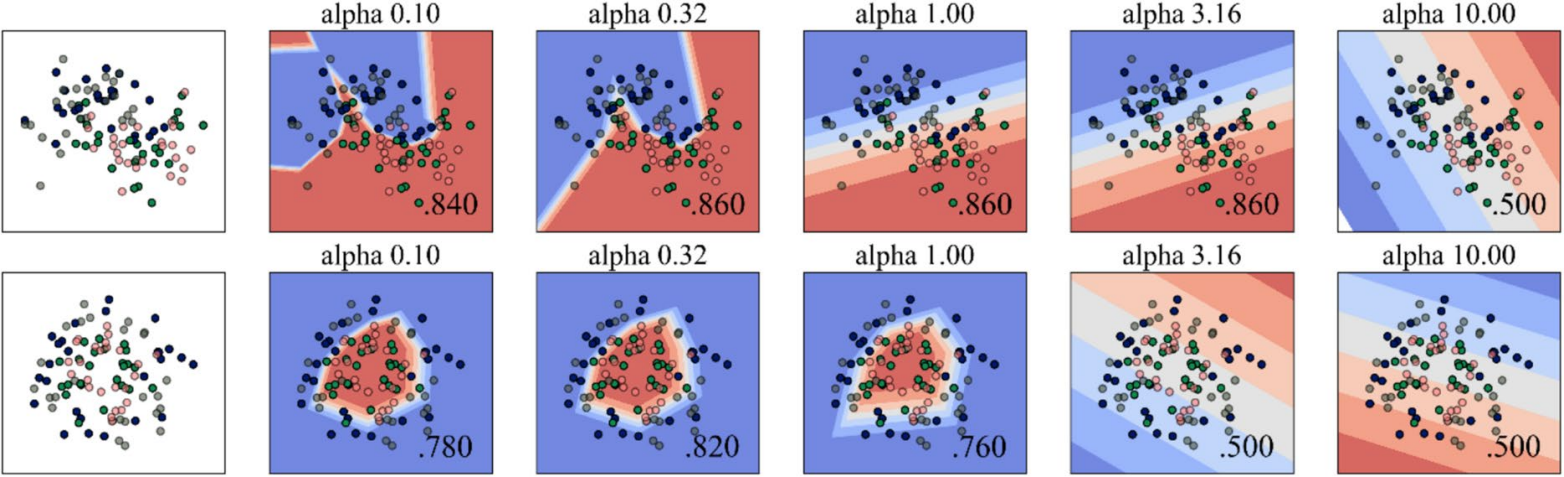
Expression of the neural model with evaluating α-values and data distribution and clustering, defining test conditions and significance level

**Fig. 8:**
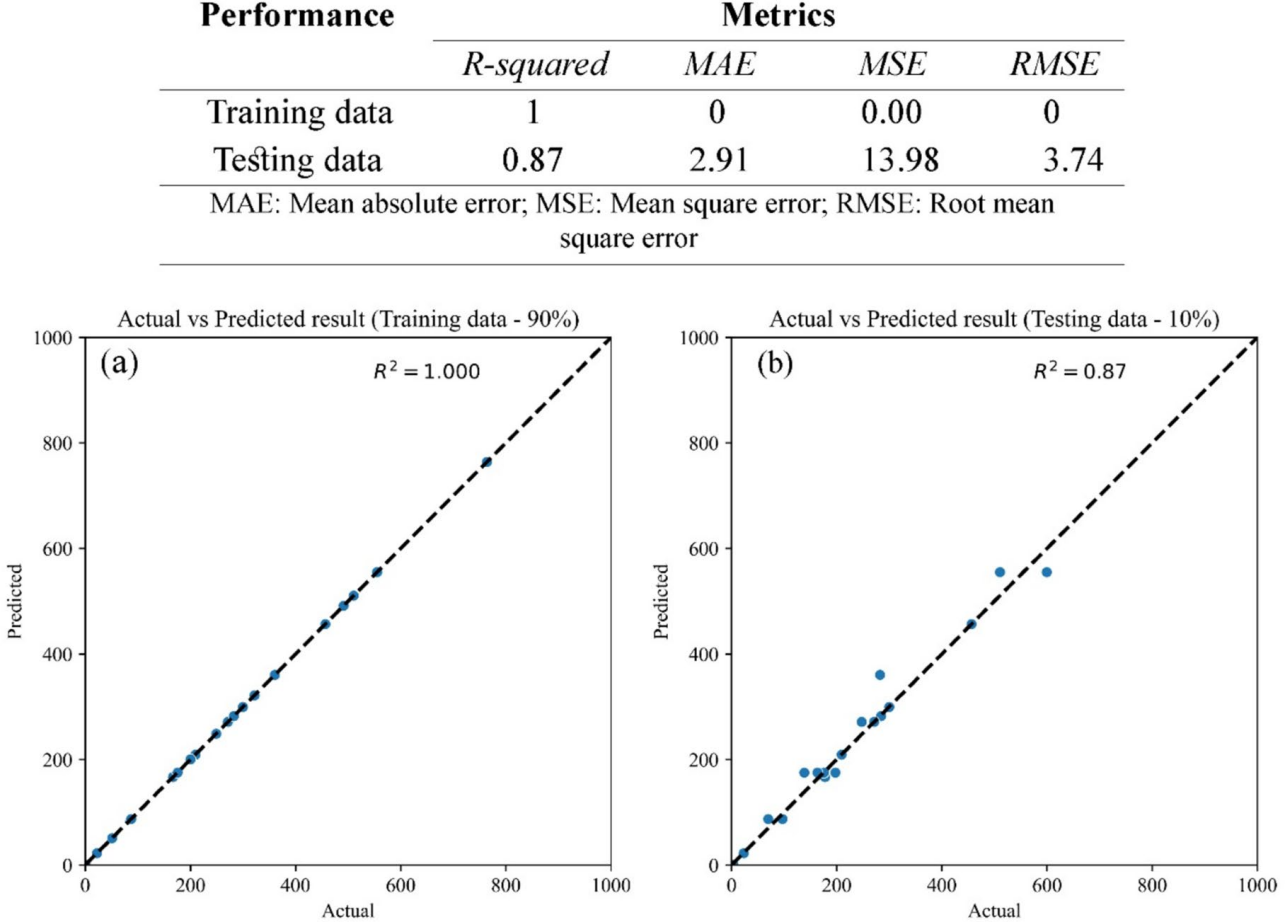
The magnitude of the model’s actions or performance while the SHAP values and influence over the output from training and testing data; Table shows the performance R^2^ = 87% for testing data size, and Fig. **a** shows actual vs predicted training data (90% data size) values, and Fig. **b** actual vs predicted testing data (10% data size) values

The model shows potential daily consumption levels of iron and arsenic in groundwater, with no pertaining risks; and the THQ = 1 value computes the safety threshold. Independent variables synthesize relationships and ɑ distribution value. This ɑ value exhibits the data’s cluster-clustered nature. It ensures the clustering procedure’s accuracy and reveals a unique underlying class within the datasets. Between the two distinct sub-groups, an 86% confidence interval (CI) is identified for α = 0.32, 1, and 3.16. Cluster 84% and 50% α = 0.10 and α = 10 in cluster 1, and even 82% CI was found at α = 0.32 in cluster 2. Based on each independent variable, ANN, and established two independent sub-groups, these data were utilized for subsequent computations (Fig. 7). The values of SHAP and their effects on the outcome are classified and examined separately for the sample sizes of 90 percent and 10 percent. Positive SHAP values have a favorable effect on forecasts; on the contrary, negative values have an adverse effect. These values’ enormity indicates the extent of their effect. 90% of the training and 10% of the tested samples couldn’t exhibit substantial variations in a feature’s contributions (Figure S2). This signifies that the SHAP values exhibit a reliable explanation of actions and efficacy of the model used to determine the secure intake threshold (Fig. 8). Higher frequency and THQ values have been considered while ascertaining how they contributed to identifying the safe intake limit. The model succeeded with 87% accuracy in simulating the condition.

**Fig. 9:**
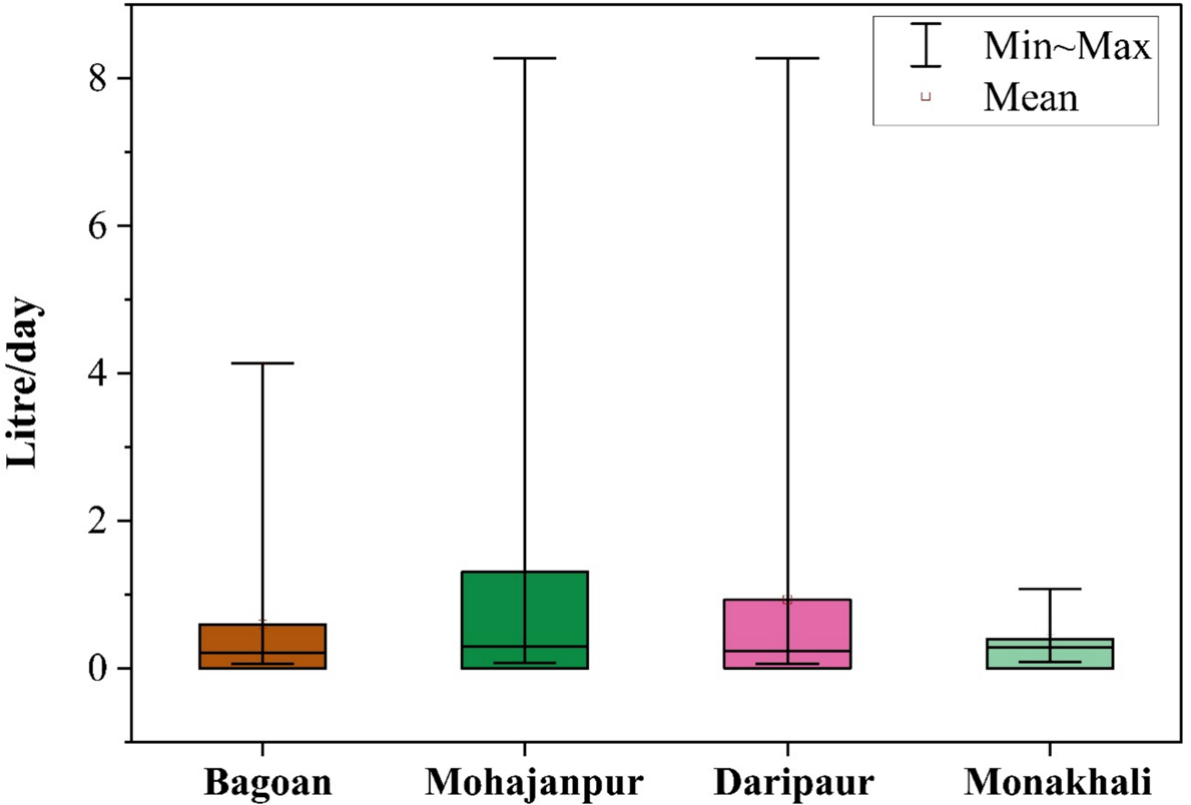
Status of the ANN-based safest water consumption limit evaluation in various locations on collected samples

The safe water consumption limits for each sampling point are presented in Table S6. The safe consumption level for Baguan Union varies from 0.06 L/day to 4.14 L/day, averaging 0.59 L/day. In Monakhali Union, the range is from 0.09 L/day to 1.08 L/day with an average of 0.39 L/day. The Moahajanpur Union has consumption limits between 0.07 L/day and 8.27 L/day, and the average value of safe water consumption is 1.30 L/day, whilst the Dariapur Union has similar limits ranging from 0.06 L/day to 8.27 L/day (Fig. 9). These thresholds are essential for safeguarding the health and safety of children, especially in regions with heightened pollutant concentrations.

## Discussion

### Groundwater quality parameters

In water samples, pH is a critical parameter in water quality evaluations since it influences the essential ions quantities such as OH^−^, CO₃^2^⁻, and HCO₃⁻ (Habib et al., 2020). The average pH was 6.85 ± 0.24 (range: 6.12–7.8), all within the permissible limit (6.5–8.5). High-quality drinking water should possess a pH range of 6.5–8.5 (WHO, 2017; US EPA, 2009; ECR, 1997). This range signifies a balanced or neutral pH in this study, establishing appropriate circumstances for consumption and adhering to health safety standards. Water samples beyond this range may signify pollution or mineral dissolution problems that could impact water quality and public health. Electrical conductivity (EC) serves as a crucial indication in this investigation, representing the dissolved ionic concentration in water. EC averaged 900.27 ± 290.48 µS/cm (range: 2.72–1767 µS/cm), exceeding the WHO limit (250 µS/cm) in 97.3% of samples, indicating high dissolved ion levels. This study’s EC readings are far beyond the acceptable threshold for drinking water quality. Prior studies have likewise reported elevated EC concentrations, corroborating these results. Rahman and Habiba (2023) indicated diminished EC values in Chuadanga municipality, implying local variations in geological or anthropogenic factors. Atikul Islam et al. (2017) asserted that the dissolution of minerals in aquifers likely results in elevated EC values by releasing ions that increase conductivity. Ingesting water with elevated EC levels is associated with negative health impacts, including renal failure risk, hypertension, and the development of intestinal calculi (Rahman & Rahman, 2018).

Further, TDS averaged 460.50 ± 144.30 mg/L (range: 1.37–882 mg/L), with 36% and 88% exceeding, respectively, the limits of US EPA (500 mg/L), WHO (600–1000 mg/L), and BDWS (1000 mg/L) standards. Elevated concentrations of TDS significantly affect water quality, bearing critical implications for health. While TDS is not intrinsically hazardous, elevated levels in water for drinking may have indirect health consequences. A substantial proportion of the samples had TDS concentrations beyond the guidelines established by the EPA and WHO. Elevated TDS levels can result in ailments such as gallstones, arterial obstructions, cardiovascular illness, and gastrointestinal disturbances, including both laxative and constipation effects (Meride & Ayenew, 2016). Nevertheless, the highest concentration of TDS is observed as a result of the long-term water–rock reactions in a deteriorating condition, industrial toxic waste, domestic sewage, and intense evapotranspiration (Chetia et al., 2012), which can influence the taste and general acceptability of drinking water.

### Correlation statistics among water quality parameters

This study revealed a strong positive association among salinity-related factors like EC and TDS. The primary cause behind the positive relationship between EC and TDS in water samples is the high concentration of dissolved ions, which simultaneously increases both conductivity and total dissolved solids. Gajendran & Thramarai (2008) evaluated a robust correlation between EC and TDS in groundwater samples from the Namibiyar River Basin, India, and Priya and Arulraj (2011) observed a similar trend in groundwater in Coimbatore City, India. This study also determined a negative association between EC and the analyzed water temperature, suggesting that higher temperatures may promote water evaporation and result in less effective ion concentration.

In contrast, the present study indicated a positive, but weak correlation (r = 0.22) between arsenic and iron. It’s implying a metal individuality and a small association between Fe and As concentrations. The positive correlation, potentially due to the geochemical processes in aquifers that allow iron oxides to adsorb arsenic. When iron minerals dissolve or are reduced, they often release arsenic into groundwater, resulting in elevated concentrations of both elements. Rahman et al. (2019) measured a favorable association (r = 0.407) between iron and arsenic in Bangladesh’s southwestern region. In another study, Rahman et al., (2021a, 2021b) identified that As and Fe are positively, but moderately, correlated in the drinking water of high school children in Bangladesh. Above, both studies are almost similar to the present study.

A similar trend was revealed by Principal Component Analysis (PCA) and confirmed that EC, TDS, and salinity clustered on both principal components’ loading with positive correlations, indicating a shared source or process. Marín Celestino et al. (2018) also found that the EC and TDS are strongly associated in the aquifer of the state of Baja California Peninsula in Mexico. PCA also confirmed that salinity-related factors dominate one principal axis, while geochemically mobilized metals dominate another, as well as the depth of the tubewell plays a crucial role for groundwater safety by acting as a main mitigating factor to eliminate the health impacts due to exposure to arsenic and iron contamination. Pandey et al. (2023), utilizing this approach, found that rock weathering and mineral leaching processes are impacted by natural and geogenic effects, which are the main causes of changes in groundwater quality. The depths of the tubewell highlighted the inverse relationship with arsenic (As) and iron (Fe) contamination in the study area.

However, both principal components’ loadings are consistent, while PC1 expressed positive, and PC2 expressed negative, and are moderately correlated within the same cluster. Among the two principal components, very weak and mixed load was observed for a subset of water quality variables, such as pH and temperature, representing their less contribution to correlate with other variables. However, PCA analysis confirmed that As and Fe loaded strongly on a different component, indicating their geochemical uniqueness. Overall, the PCA illustrates a clear separation of influential parameters along PC1 and PC2, allowing for efficient grouping and comparison of sampling sites based on multivariate water quality characteristics. This suggests that the study areas can be differentiated effectively based on a subset of water quality variables, enabling targeted environmental monitoring and management strategies.

The decrease in iron and arsenic concentrations with increasing tubewell depth is primarily due to the natural filtering effects of deeper geological layers, which reduce the presence of these elements. Hossain et al. (2023) showed a strong negative correlation between Fe concentration and depth by applying the Pearson correlation coefficient, concluding that Fe concentration declines with increasing depth. Islam et al. (2023) observed a similar pattern, stating that increasing tubewell depths will decrease arsenic concentrations. So, this integrated approach suggests the influences of salinity-related factors and metal mobilization on the groundwater quality.

### Health risks and recommendations for safe water consumption

Health risk assessment and the safe water consumption limits have been evaluated based on iron (Fe) and arsenic (As) concentrations. Table 3 expresses recent data on Fe and As concentrations, with the present study findings in tubewell water across various regions of Bangladesh. Fe levels are notably high in Sylhet (up to 15.95 mg/L) and Panchagar (up to 10 mg/L), while much lower levels are observed in Kushtia (range 0.04–1.45 mg/L). As concentrations vary widely, with the highest levels found in Rangpur (up to 0.994 mg/L) and Satkhira (up to 0.25 mg/L), and the lowest in Lalmonirhat and Khulna, only up to 0.01 and 0.0035 mg/L, respectively (Ahmed et al., 2019; Rahman & Rahaman, 2018; Rahman et al., 2021a, 2021b). Groundwater Fe and As levels show notable regional variation, with elevated iron in Sylhet and high As in Rangpur, indicating potential local contamination sources. In this study, Fe averaged 0.94 ± 0.92 mg/L, surpassing WHO and US EPA limits, e.g., 0.3 mg/L in 68%, and BDWS, e.g., 0.3–1.0 mg/L in 32%. As averaged 0.03 ± 0.05 mg/L and ranged from 0 to 0.175 mg/L, exceeding WHO/US EPA, e.g., 0.01 mg/L in 48%, and BDWS, e.g., 0.05 mg/L in 26.7%. As concentrations in the Dariapur and Mohajanpur unions are 0.046 and 0.021 mg/L, respectively. In contrast, the tubewell water for both the Bagoan and Monakhali unions contained 0.047 and 0.0.24 mg/L of As, respectively. Compared to the global research, the concentration of Fe in Mujibnagar is markedly lower than in the USA (up to 25.0 mg/L) and Ghana (up to 13.7 mg/L) (Acquah et al., 2025; Adeloju et al., 2021), and is consistent with the levels reported in Nepal, Finland, and India (Aswal et al., 2023; Luoma et al., 2024; Sarkar et al., 2022). As concentrations are comparatively low, remaining below the worldwide maxima of 2.6 mg/L in Nepal and 0.6 mg/L in Italy (Sarkar et al., 2022; Triassi et al., 2023). The findings indicate a comparatively moderate level of Fe and As pollution in the groundwater of Mujibnagar compared to various worldwide hotspots (Table 3).

**Table 3:** Hazard Quotient (HQ) range of iron (Fe) and arsenic (As) in groundwater samples from tested union

Sampling sites	Total sample	Hazard quotient (HQ)				Hazard indices (HI)
		Iron (Fe)		Arsenic (As)		
		Conc. range (mg/L)	HQ > 1	Conc. range (mg/L)	HQ > 1	Ranges
Bagoan	29	0 – 2.76	n = 26 (1.24 – 26.29)	0 – 0.175	n = 14 (7.78 – 38.89)	0.57 – 60.13
Monakhali	15	0.23–2.72	n = 15 (2.19 – 25.9)	0 – 0.075	n = 7 (7.78 – 16.67)	2.19 – 30.54
Moahajanpur	17	0 – 3.66	n = 15 (1.62 – 34.86)	0 – 0.075	n = 8 (7.78 – 16.67)	0 – 34.86
Dariapur	14	0 – 3.38	n = 11 (1.43 – 32.19)	0 – 0.175	n = 7 (5.56 – 38.89)	0 –71.08

This study also revealed notable variations of HQ and HI across the study area. Due to continuous drinking of groundwater in the study area’s inhabitants may experience several non-carcinogenic health hazards, such as injuries to the skin, vomiting, abdominal pain, diarrhoea, respiratory tract, and gastrointestinal infections, liver damage, hematopoietic, nervous system, cardiovascular, neurological and reproductive problems, diabetes, and hair loss (Chakraborty et al., 2022). The various trace metal(loid)s found in drinking water might have serious impacts on adults and children. For humans, a moderate amount of Fe is necessary, but high or very low amounts of Fe may cause various problems. For example, low Fe concentration results in anaemia in adults and children in developing nations and changes the myelination, neurotransmission, metabolism, and protein and gene profiles of the brain (Beinner et al., 2005; Walker et al., 2007). On the contrary, excessive Fe can have negative effects on water quality, e.g., lower dissolved oxygen (DO), and the health of human beings, e.g., kidney stones. Children are susceptible to pediatric pneumonia, respiratory symptoms, cardiovascular disorders, and lung cancers, kidneys, and bladder because of consuming water from these sources (Osorio-Ya´n˜ez et al., 2013; Rahman et al., 2013a, 2013b; Osorio-Ya´n˜ez et al., 2015; Smith et al., 2013). As arsenic enters the placenta easily, various epidemiological investigations showed that exposure to arsenic may have a moderately elevated risk of prenatal development impairment and increased prenatal and infant mortality. Rahman et al., (2013a, 2013b) found that arsenic exposure is associated with a greater chance of infant mortality. Welch et al. (2019) stated that prenatal arsenic exposure might have a detrimental effect on the immunologic development of early childhood in Bangladesh. George et al. (2015) identified that urinary arsenic concentration is related to an elevated risk of pneumonia, suggesting that a low to moderate arsenic exposure might be a risk indicator for pneumonia among children, specifically those below five years old. Rahman et al. (2017) also discovered that exposure to As has been linked to a higher occurrence of diarrhoea and infection of the respiratory tract among children. According to epidemiological studies, it is postulated that there might be relationships between As exposure, and anaemia in children may result in growth retardation (Gardner et al., 2013; Heck et al., 2008; Sazawal et al., 2010).

The assessment of safe consumption limits for tubewell water contaminated with iron and arsenic has disclosed substantial variations contingent upon the contamination level. This analysis demonstrated that the samples’ A sconcentrations consisted of a mean of 0.037 mg/L, and ranged from 0.000 to 0.175 mg/L. The Fe levels were more diverse, with an average of 0.949 mg/L and a range of 0.000–3.660 mg/L. The results emphasize the significant spatial variations in safe groundwater consumption among different unions in Mujibnagar, Bangladesh, which suggest potential health hazards for school children and water quality concerns. The Baguan and Monakhali unions have comparatively lower safe consumption limits, with averages of 0.59 and 0.39 L/day, respectively. This suggests that the health risks are higher if the contamination levels are increased. The Moahajanpur and Dariapur Unions, in contrast, exhibit broader safe consumption ranges, the highest average (1.30 L/day) in Moahajanpur, which suggests that the water purity is superior or the health risks are minimal. These modeling-based findings highlight the serious health risks associated with elevated  Fe and As levels in drinking water. Chronic As exposure, even at low concentrations, is linked to cancer, neurological issues, and cardiovascular disease (WHO, 2010; Smith et al., 2000), while excessive iron can cause gastrointestinal distress and oxidative damage (WHO, 2011). Over 68% of samples exceeded WHO limits (0.01 mg/L for As; 0.3 mg/L for Fe), underscoring the urgent need for targeted interventions, including improved filtration, monitoring, and public awareness. The observed trends highlight the importance of integrating site-specific data into broader water safety models to better guide risk mitigation efforts. These findings contribute meaningfully to the growing body of research on groundwater contamination in developing regions and offer valuable insights for advancing sustainable water management practices to save people’s health (Chakraborti et al., 2017; Rahman et al., 2021b).

### Limitations and prospective research directions

This study evaluated the groundwater quality of the primary school in Mujibnagar Upazila, including the children’s health risks. It quantified the safe water consumption limit for schoolgoers to avoid adverse effects on health. Based on the findings of this study, shallow water contains higher iron and arsenic concentrations, posing health risks. Besides promising findings, this study has evaluated some limitations, such as utilizing the handheld iron checker (Hanna HI-721), which was only able to express the iron concentration (0.00–5.00 mg/L). Similarly, the Hatch-Hach EZ Dual-Range Arsenic Tester only provides the concentration range without any fractional number between 0.00 and 5.00 mg/L. Those limits of detection may sometimes affect accuracy and high-range measurements. Future studies should incorporate advanced laboratory-based analytical techniques, such as Inductively Coupled Plasma Mass Spectrometry (ICP-MS), Atomic Absorption Spectroscopy (AAS), or Inductively Coupled Plasma Optical Emission Spectrometry (ICP-OES) are recommended for precise and accurate quantification of trace metals. These techniques provide lower detection limits, higher sensitivity, and the ability to measure a broader concentration range with greater precision, thereby improving data quality and enabling a more robust assessment of metal contamination levels. In addition, the tubewells’ depth information was collected from the schools’ authorities, which may have been slightly biased or lacked solid information. In contrast, we measured arsenic and iron concentration data during pre-monsoon conditions, not considering seasonal variation in the concentrations of contaminants. To enhance the accuracy and reliability of the findings, future studies should aim to obtain precise well-depth data through direct field measurement or official geological records rather than relying solely on secondary information from school authorities. Additionally, arsenic and iron concentration measurements should be conducted across multiple seasons (e.g., pre-monsoon, monsoon, and post-monsoon) to account for potential seasonal variations in contaminant levels and provide a more comprehensive understanding of groundwater quality dynamics. Further, the potential health risks have been estimated, focusing primarily on iron and arsenic contamination, not considering other possible heavy metal contamination. Future studies should aim to expand this research to a wider regional or national scale, covering various upazilas or districts and including a full spectrum of heavy metals and microbial contaminants. Furthermore, future analyses would allow for a more holistic assessment of the nutritional status of drinking water, including key elements such as Na⁺, Mg^2^⁺, Ca^2^⁺, Mn^2^⁺, and Zn^2^⁺, which are essential for both human health and environmental functions, should also be considered. This includes evaluating the Drinking Water Nutritional Quality Index (DWNQI) in conjunction with region-specific geoscience data to generate actionable insights for water resource management and to support the achievement of Sustainable Development Goals (SDGs). A similar approach was proposed by Agbasi et al., (2024, 2025b). Overall, these efforts would support the development of comprehensive risk maps and region-specific management strategies for school tubewell water across Bangladesh, with potential for global implementation.

## Summary and conclusion

This study assessed the health risks to children from groundwater contamination in Mujibnagar Upazila, Kushtia, focusing on iron (Fe) and arsenic (As) levels in daily tubewell water consumption. The analysis revealed that concentrations of both iron and arsenic frequently exceeded permissible limits, with 68% of samples surpassing water quality standards for iron and 48% for arsenic, as defined by the WHO and US EPA. In comparison, 32% and 26.7% of samples exceeded the Bangladesh Drinking Water Standards (BDWS) for iron and arsenic, respectively. Electrical conductivity and total dissolved solids were consistently high, indicating significant salinity-related concerns. A critical finding is the inverse relationship between tubewell depth and contaminant concentration, underscoring the protective role of deeper aquifers. The map of spatial distribution and HQ (hazard quotient) revealed that Monakhali is the most vulnerable union compared with others because of the higher levels of iron and arsenic. The total dissolved solid (TDS) was strongly correlated with conductivity (r = 0.92) and moderately with salinity (r = 0.7), highlighting their mutual association. A weak positive correlation was observed between As and Fe. Principal components PC1 and PC2 accounted for 56.74% of the total variance; while Fe and As showed weak correlation in PC1, both had negative loadings in PC2, reflecting differing patterns shaped by TDS, conductivity, and salinity. Additionally, linear regression revealed declining trends of Fe and As (with increasing depth. Based on Artificial Neural Network (ANN) evaluation, the Monakhali area estimated the lower acceptable tubewell water consumption rate (average 0.39 L/day). Conversely, the Mohajanpur union is the safest area, and is estimated to have the highest safe consumption limit (average 1.30 L/day), indicating the lower concentration levels of Fe and As in the tubewell water of this school. However, few places are estimated to have lower safe limits. The potential utilisation of ANN modeling to accumulate other metals or heavy metalloids will provide a more comprehensive and robust study, as well as evaluate critical public health issues and sustainable solutions to safe consumption limits of tubewell water across the study region and beyond.

## Supplementary Information

Below is the link to the electronic supplementary material.

Supplementary file1 (DOCX 196 KB)

## Data Availability

Supplementary data have been added.
