# Supplementary material for: Groundwater quality assessment and health risk evaluation for schoolchildren in Mujibnagar, Bangladesh: safe consumption guidelines using artificial neural network modeling
Source: Environ Geochem Health. 2025 Jul 20;47(8):324. doi: 10.1007/s10653-025-02627-1 (PMC12277229; doi:10.1007/s10653-025-02627-1)
Supplement: Supplementary file 1 — Supplementary file1 (DOCX 196 KB) [file 10653_2025_2627_MOESM1_ESM.docx]

**Groundwater Quality Assessment and Health Risk Evaluation for Schoolchildren in Mujibnagar, Bangladesh: Safe Consumption Guidelines using Artificial Neural Network Modeling**

Mohammad Omar Faruk Molla^1^, Md. Anisul Kabir^1^, Md. Kamrul Hossain^1^, Md. Saikoth Jahan^1^, Most. Suria Khatun^1^, Sazal Kumar^3^, Rafiquel Islam^2, 3*^

*^1^Department of Geography and Environment (GE), Islamic University, Kushtia-7003, Bangladesh*

*^2^Department of Applied Chemistry and Chemical Engineering (ACCE), Islamic University, Kushtia-7003, Bangladesh*

*^3^School of Environmental and Life Sciences, The University of Newcastle (UoN), Callaghan, NSW 2308, Australia*

****Corresponding Author***

Dr. Rafiquel Islam, E-mail: rafiq@acce.iu.ac.bd; [rafiquel.islam@newcastle.edu.au](mailto:rafiquel.islam@newcastle.edu.au)

ORCID: 0000-0002-3033-4840

**Table S1** Methods and equipment used in water quality parameters analysis

| **Parameters** | **Materials/Equipment** | **Unit** |
| --- | --- | --- |
| Iron (Fe) | Hanna HI-721 Iron Checker Handheld Colorimeter | mg/l |
| Arsenic (As) | Hach EZ Dual-Range Arsenic Test Kit | mg/l |
| Temperature | Portable Glass Mercury Thermometer | (^0^C) |
| pH | Model EZD0-6011 | ** |
| Salinity | Water Quality Tester (Five in One) | ppm |
| TDS | Ezdo TDS-5031 | ppm |
| EC | OHAUS Conductivity Portable Meter | µs/cm |

**Table S2** Details information of analyzed water sample

| **Sample Number** | **Latitude** | **Longitude** | **Union Name** | **As (mg/l)** | **Fe (mg/l)** | **Depths (feet)** | **TDS (ppm)** | **Salinity (ppm)** | **EC (µs/cm)** | **pH** | **Temperature (⁰C)** |
| --- | --- | --- | --- | --- | --- | --- | --- | --- | --- | --- | --- |
| 1 | 23.648502 | 88.596327 | Baguan | 0.075 | 0.18 | 250 | 489 | 635 | 982 | 6.7 | 26.2 |
| 2 | 23.639862 | 88.612887 | Baguan | 0 | 0.46 | 560 | 469 | 610 | 938 | 6.85 | 26.5 |
| 3 | 23.631013 | 88.609684 | Baguan | 0.075 | 1.56 | 120 | 333 | 433 | 657 | 6.89 | 26.4 |
| 4 | 23.624835 | 88.606197 | Baguan | 0 | 0.47 | 450 | 424 | 549 | 843 | 6.75 | 26.7 |
| 5 | 23.636144 | 88.611614 | Baguan | 0 | 1 | 250 | 520 | 679 | 1037 | 6.64 | 26.3 |
| 6 | 23.631946 | 88.612408 | Baguan | 0.175 | 0.51 | 100 | 319 | 414 | 635 | 6.96 | 26.1 |
| 7 | 23.632272 | 88.612343 | Baguan | 0 | 1.35 | 350 | 596 | 777 | 1191 | 6.59 | 26.2 |
| 8 | 23.652588 | 88.587699 | Baguan | 0 | 0.14 | 450 | 405 | 526 | 823 | 6.86 | 26.8 |
| 9 | 23.646847 | 88.592721 | Baguan | 0 | 0.88 | 550 | 388 | 505 | 764 | 6.83 | 26.3 |
| 10 | 23.664851 | 88.604082 | Baguan | 0.075 | 1.99 | 120 | 814 | 1060 | 1624 | 6.96 | 25.8 |
| 11 | 23.642315 | 88.581003 | Baguan | 0.175 | 0.59 | 100 | 738 | 959 | 1466 | 6.98 | 26.7 |
| 12 | 23.648486 | 88.583913 | Baguan | 0 | 0.13 | 400 | 470 | 611 | 937 | 6.82 | 26.5 |
| 13 | 23.662149 | 88.581365 | Baguan | 0 | 0.2 | 300 | 579 | 753 | 1153 | 6.83 | 26.4 |
| 14 | 23.664209 | 88.603041 | Baguan | 0.075 | 0 | 120 | 527 | 683 | 1047 | 6.85 | 26.3 |
| 15 | 23.658647 | 88.613858 | Baguan | 0 | 0.59 | 370 | 434 | 565 | 868 | 6.81 | 26.7 |
| 16 | 23.660962 | 88.616535 | Baguan | 0.075 | 0.78 | 150 | 539 | 700 | 1073 | 6.75 | 25.3 |
| 17 | 23.64849 | 88.650612 | Baguan | 0 | 0.35 | 500 | 473 | 615 | 949 | 6.74 | 27.1 |
| 18 | 23.622362 | 88.626736 | Baguan | 0 | 0.81 | 560 | 457 | 592 | 917 | 6.83 | 27.3 |
| 19 | 23.61873 | 88.624975 | Baguan | 0 | 0.66 | 560 | 367 | 478 | 729 | 7.12 | 26.1 |
| 20 | 23.611096 | 88.623277 | Baguan | 0.175 | 2.23 | 120 | 396 | 515 | 788 | 6.72 | 26.8 |
| 21 | 23.620861 | 88.606326 | Baguan | 0.075 | 0.17 | 350 | 1.37 | 1.79 | 2.72 | 6.44 | 26.9 |
| 22 | 23.650022 | 88.647339 | Baguan | 0 | 0.09 | 270 | 340 | 445 | 680 | 7.01 | 27 |
| 23 | 23.648379 | 88.643196 | Baguan | 0.175 | 0.15 | 120 | 632 | 822 | 1261 | 6.77 | 26.9 |
| 24 | 23.621014 | 88.617398 | Baguan | 0 | 0.74 | 560 | 530 | 976 | 712 | 6.83 | 27.8 |
| 25 | 23.623057 | 88.622459 | Baguan | 0.035 | 1.21 | 150 | 362 | 1237 | 953 | 6.79 | 26.3 |
| 26 | 23.615009 | 88.622601 | Baguan | 0.075 | 2.5 | 100 | 430 | 562 | 690 | 6.53 | 27.6 |
| 27 | 23.64359 | 88.587183 | Baguan | 0 | 0.06 | 350 | 542 | 694 | 1240 | 6.72 | 25.8 |
| 28 | 23.623901 | 88.616447 | Baguan | 0.035 | 2.4 | 150 | 270 | 808 | 980 | 6.98 | 23.9 |
| 29 | 23.6548 | 88.590563 | Baguan | 0.075 | 2.76 | 220 | 478 | 890 | 870 | 6.87 | 24.7 |
| 30 | 23.675671 | 88.608209 | Monakhali | 0 | 2.72 | 150 | 382 | 495 | 762 | 7.03 | 27.2 |
| 31 | 23.674996 | 88.613496 | Monakhali | 0.075 | 0.8 | 250 | 330 | 430 | 660 | 7.14 | 26.8 |
| 32 | 23.670679 | 88.603599 | Monakhali | 0 | 0.36 | 405 | 463 | 601 | 926 | 6.93 | 26.8 |
| 33 | 23.673272 | 88.615454 | Monakhali | 0.035 | 1.32 | 320 | 497 | 647 | 996 | 6.77 | 26.9 |
| 34 | 23.668298 | 88.618288 | Monakhali | 0 | 0.23 | 370 | 443 | 575 | 886 | 6.75 | 26.8 |
| 35 | 23.677033 | 88.615477 | Monakhali | 0.035 | 0.28 | 300 | 415 | 538 | 829 | 6.95 | 26.5 |
| 36 | 23.710009 | 88.613214 | Monakhali | 0.035 | 2.39 | 320 | 433 | 562 | 870 | 6.87 | 26.4 |
| 37 | 23.713613 | 88.613431 | Monakhali | 0 | 0.26 | 370 | 780 | 1082 | 1654 | 6.71 | 26.5 |
| 38 | 23.676632 | 88.604662 | Monakhali | 0 | 0.23 | 370 | 284 | 368 | 565 | 7.07 | 26.8 |
| 39 | 23.652286 | 88.636257 | Monakhali | 0.075 | 0.69 | 220 | 431 | 562 | 864 | 6.87 | 26.4 |
| 40 | 23.68943 | 88.676368 | Monakhali | 0 | 1.87 | 370 | 473 | 613 | 936 | 6.8 | 27.9 |
| 41 | 23.674027 | 88.621861 | Monakhali | 0 | 0.7 | 370 | 340 | 958 | 882 | 6.73 | 25.9 |
| 42 | 23.670531 | 88.610899 | Monakhali | 0.075 | 0.24 | 200 | 410 | 1324 | 786 | 6.12 | 26.6 |
| 43 | 23.674468 | 88.610654 | Monakhali | 0.035 | 0.57 | 320 | 370 | 541 | 540 | 7.12 | 27.3 |
| 44 | 23.677811 | 88.612818 | Monakhali | 0 | 0.87 | 370 | 423 | 532 | 670 | 6.88 | 28.9 |
| 45 | 23.66043 | 88.652199 | Moahajanpur | 0 | 0 | 370 | 611 | 793 | 1227 | 6.74 | 26.5 |
| 46 | 23.676637 | 88.667654 | Moahajanpur | 0 | 0.5 | 370 | 331 | 430 | 656 | 7.03 | 26.5 |
| 47 | 23.683435 | 88.674885 | Moahajanpur | 0.035 | 1.89 | 320 | 435 | 565 | 871 | 6.29 | 27.9 |
| 48 | 23.681008 | 88.677897 | Moahajanpur | 0.075 | 0.25 | 150 | 506 | 658 | 1010 | 6.84 | 27.9 |
| 49 | 23.652602 | 88.66817 | Moahajanpur | 0 | 0.3 | 370 | 353 | 458 | 700 | 6.93 | 27.7 |
| 50 | 23.653969 | 88.673069 | Moahajanpur | 0.035 | 0.53 | 320 | 1.45 | 1.88 | 2.87 | 6.66 | 28.2 |
| 51 | 23.650447 | 88.672408 | Moahajanpur | 0 | 3.66 | 370 | 536 | 700 | 1066 | 6.78 | 26.3 |
| 52 | 23.65803 | 88.677425 | Moahajanpur | 0 | 0.17 | 370 | 443 | 575 | 891 | 6.72 | 28.15 |
| 53 | 23.680091 | 88.68304 | Moahajanpur | 0.035 | 0.83 | 405 | 531 | 690 | 1058 | 6.77 | 27.8 |
| 54 | 23.700003 | 88.6819 | Moahajanpur | 0.075 | 0.5 | 220 | 504 | 657 | 999 | 6.77 | 28 |
| 55 | 23.696163 | 88.675269 | Moahajanpur | 0 | 0.39 | 370 | 511 | 659 | 1020 | 6.9 | 26.8 |
| 56 | 23.692467 | 88.674179 | Moahajanpur | 0 | 0.5 | 370 | 336 | 438 | 667 | 6.87 | 27.6 |
| 57 | 23.65285 | 88.677082 | Moahajanpur | 0.035 | 1.34 | 405 | 563 | 628 | 780 | 7.29 | 27.9 |
| 58 | 23.655599 | 88.675297 | Moahajanpur | 0 | 1.27 | 370 | 390 | 734 | 794 | 6.78 | 24.8 |
| 59 | 23.653159 | 88.677802 | Moahajanpur | 0 | 0.03 | 370 | 654 | 869 | 870 | 7.78 | 26.7 |
| 60 | 23.687003 | 88.676586 | Moahajanpur | 0.035 | 2.15 | 320 | 572 | 1010 | 934 | 6.89 | 25.7 |
| 61 | 23.685243 | 88.674347 | Moahajanpur | 0.035 | 1.7 | 405 | 483 | 987 | 975 | 7.32 | 28.7 |
| 62 | 23.681052 | 88.610066 | Dariapur | 0 | 0.97 | 370 | 464 | 602 | 919 | 6.7 | 27.8 |
| 63 | 23.691663 | 88.609843 | Dariapur | 0 | 0 | 405 | 469 | 609 | 936 | 6.97 | 27.6 |
| 64 | 23.700568 | 88.604183 | Dariapur | 0 | 0.23 | 370 | 351 | 457 | 701 | 6.73 | 27.8 |
| 65 | 23.703873 | 88.600354 | Dariapur | 0.035 | 1.11 | 305 | 416 | 544 | 826 | 7 | 27.5 |
| 66 | 23.709378 | 88.609769 | Dariapur | 0 | 2.25 | 120 | 512 | 665 | 1023 | 6.76 | 27 |
| 67 | 23.709331 | 88.607583 | Dariapur | 0.175 | 3.38 | 120 | 882 | 1146 | 1767 | 6.73 | 28 |
| 68 | 23.70175 | 88.5996 | Dariapur | 0.035 | 0.45 | 320 | 559 | 725 | 1117 | 6.84 | 27.4 |
| 69 | 23.707921 | 88.609099 | Dariapur | 0.175 | 0 | 150 | 506 | 658 | 1012 | 6.88 | 27.4 |
| 70 | 23.70618 | 88.594986 | Dariapur | 0 | 1.36 | 370 | 354 | 459 | 704 | 6.9 | 27.4 |
| 71 | 23.710226 | 88.596147 | Dariapur | 0 | 0.15 | 370 | 736 | 955 | 1449 | 6.88 | 25.9 |
| 72 | 23.711378 | 88.610055 | Dariapur | 0.025 | 0.07 | 360 | 612 | 1357 | 1270 | 7.23 | 24.9 |
| 73 | 23.709668 | 88.610884 | Dariapur | 0.035 | 2.8 | 200 | 286 | 629 | 568 | 6.76 | 25.9 |
| 74 | 23.709605 | 88.605356 | Dariapur | 0.175 | 3.1 | 150 | 322 | 544 | 574 | 7.8 | 27.8 |
| 75 | 23.711377 | 88.607441 | Dariapur | 0 | 0.83 | 500 | 512 | 665 | 687 | 6.76 | 26.9 |

**Table S3:** PCA analysis using all water quality parameters across the study areas

| **PCN** | **Parameters** | **Eigenvalues** | **PV (%)** | **Cumulative (%** | **PC1** | **PC2** |
| --- | --- | --- | --- | --- | --- | --- |
|  |  |  |  |  | ***Loadings*** | |
| 1 | As | 2.76878 | 34.60971 | 34.60971 | 0.214 | -0.589 |
| 2 | Fe | 1.77061 | 22.13259 | 56.7423 | 0.136 | -0.391 |
| 3 | Depths | 1.04491 | 13.06143 | 69.80373 | -0.248 | 0.6068 |
| 4 | TDS | 0.97908 | 12.23844 | 82.04218 | 0.524 | 0.2369 |
| 5 | Salinity | 0.81131 | 10.14137 | 92.18355 | 0.508 | 0.1739 |
| 6 | Conductivity | 0.31414 | 3.92674 | 96.11029 | 0.5464 | 0.208 |
| 7 | pH | 0.25132 | 3.14151 | 99.2518 | 0.0117 | -0.035 |
| 8 | Temperature | 0.05986 | 0.7482 | 100 | -0.2025 | -0.026 |
| PCN: Principal Component Number; PV: Percentage of Variance | | | | | | |

**Table S4**: HQ and HI values of iron (Fe) and arsenic (As)

| **Sample number** | **Union name** | **Conc. (As)** | **CDI (As)** | **Rfd (As)** | **HQ (As)** | **Conc. (Fe)** | **CDI (Fe)** | **Rfd (Fe)** | **HQ (Fe)** | **HI** |
| --- | --- | --- | --- | --- | --- | --- | --- | --- | --- | --- |
| 1 | Baguan | 0.075 | 0.005 | 0.0003 | 16.667 | 0.18 | 0.012 | 0.007 | 1.714 | 18.381 |
| 2 | Baguan | 0 | 0 | 0.0003 | 0 | 0.46 | 0.031 | 0.007 | 4.381 | 4.381 |
| 3 | Baguan | 0.075 | 0.005 | 0.0003 | 16.667 | 1.56 | 0.104 | 0.007 | 14.857 | 31.523 |
| 4 | Baguan | 0 | 0 | 0.0003 | 0 | 0.47 | 0.031 | 0.007 | 4.476 | 4.476 |
| 5 | Baguan | 0 | 0 | 0.0003 | 0 | 1 | 0.067 | 0.007 | 9.524 | 9.524 |
| 6 | Baguan | 0.175 | 0.012 | 0.0003 | 38.889 | 0.51 | 0.034 | 0.007 | 4.857 | 43.746 |
| 7 | Baguan | 0 | 0 | 0.0003 | 0 | 1.35 | 0.09 | 0.007 | 12.857 | 12.857 |
| 8 | Baguan | 0 | 0 | 0.0003 | 0 | 0.14 | 0.009 | 0.007 | 1.333 | 1.333 |
| 9 | Baguan | 0 | 0 | 0.0003 | 0 | 0.88 | 0.058 | 0.007 | 8.381 | 8.381 |
| 10 | Baguan | 0.075 | 0.005 | 0.0003 | 16.667 | 1.99 | 0.133 | 0.007 | 18.952 | 35.619 |
| 11 | Baguan | 0.175 | 0.012 | 0.0003 | 38.889 | 0.59 | 0.039 | 0.007 | 5.619 | 44.508 |
| 12 | Baguan | 0 | 0 | 0.0003 | 0 | 0.13 | 0.008 | 0.007 | 1.238 | 1.238 |
| 13 | Baguan | 0 | 0 | 0.0003 | 0 | 0.2 | 0.013 | 0.007 | 1.905 | 1.905 |
| 14 | Baguan | 0.075 | 0.005 | 0.0003 | 16.667 | 0 | 0 | 0.007 | 0 | 16.667 |
| 15 | Baguan | 0 | 0 | 0.0003 | 0 | 0.59 | 0.039 | 0.007 | 5.619 | 5.619 |
| 16 | Baguan | 0.075 | 0.005 | 0.0003 | 16.667 | 0.78 | 0.052 | 0.007 | 7.429 | 24.095 |
| 17 | Baguan | 0 | 0 | 0.0003 | 0 | 0.35 | 0.023 | 0.007 | 3.333 | 3.333 |
| 18 | Baguan | 0 | 0 | 0.0003 | 0 | 0.81 | 0.054 | 0.007 | 7.714 | 7.714 |
| 19 | Baguan | 0 | 0 | 0.0003 | 0 | 0.66 | 0.044 | 0.007 | 6.286 | 6.286 |
| 20 | Baguan | 0.175 | 0.012 | 0.0003 | 38.889 | 2.23 | 0.149 | 0.007 | 21.238 | 60.127 |
| 21 | Baguan | 0.075 | 0.005 | 0.0003 | 16.667 | 0.17 | 0.011 | 0.007 | 1.619 | 18.286 |
| 22 | Baguan | 0 | 0 | 0.0003 | 0 | 0.09 | 0.006 | 0.007 | 0.857 | 0.857 |
| 23 | Baguan | 0.175 | 0.012 | 0.0003 | 38.889 | 0.15 | 0.01 | 0.007 | 1.429 | 40.317 |
| 24 | Baguan | 0 | 0 | 0.0003 | 0 | 0.74 | 0.049 | 0.007 | 7.048 | 7.048 |
| 25 | Baguan | 0.035 | 0.002 | 0.0003 | 7.778 | 1.21 | 0.081 | 0.007 | 11.524 | 19.302 |
| 26 | Baguan | 0.075 | 0.005 | 0.0003 | 16.667 | 2.5 | 0.167 | 0.007 | 23.809 | 40.476 |
| 27 | Baguan | 0 | 0 | 0.0003 | 0 | 0.06 | 0.004 | 0.007 | 0.571 | 0.571 |
| 28 | Baguan | 0.035 | 0.002 | 0.0003 | 7.778 | 2.4 | 0.16 | 0.007 | 22.857 | 30.635 |
| 29 | Baguan | 0.075 | 0.005 | 0.0003 | 16.667 | 2.76 | 0.184 | 0.007 | 26.286 | 42.952 |
| 30 | Monakhali | 0 | 0 | 0.0003 | 0 | 2.72 | 0.181 | 0.007 | 25.905 | 25.905 |
| 31 | Monakhali | 0.075 | 0.005 | 0.0003 | 16.667 | 0.8 | 0.053 | 0.007 | 7.619 | 24.286 |
| 32 | Monakhali | 0 | 0 | 0.0003 | 0 | 0.36 | 0.024 | 0.007 | 3.429 | 3.429 |
| 33 | Monakhali | 0.035 | 0.002 | 0.0003 | 7.778 | 1.32 | 0.088 | 0.007 | 12.571 | 20.349 |
| 34 | Monakhali | 0 | 0 | 0.0003 | 0 | 0.23 | 0.015 | 0.007 | 2.19 | 2.19 |
| 35 | Monakhali | 0.035 | 0.002 | 0.0003 | 7.778 | 0.28 | 0.019 | 0.007 | 2.667 | 10.444 |
| 36 | Monakhali | 0.035 | 0.002 | 0.0003 | 7.778 | 2.39 | 0.159 | 0.007 | 22.761 | 30.539 |
| 37 | Monakhali | 0 | 0 | 0.0003 | 0 | 0.26 | 0.017 | 0.007 | 2.476 | 2.476 |
| 38 | Monakhali | 0 | 0 | 0.0003 | 0 | 0.23 | 0.015 | 0.007 | 2.19 | 2.19 |
| 39 | Monakhali | 0.075 | 0.005 | 0.0003 | 16.667 | 0.69 | 0.046 | 0.007 | 6.571 | 23.238 |
| 40 | Monakhali | 0 | 0 | 0.0003 | 0 | 1.87 | 0.125 | 0.007 | 17.809 | 17.809 |
| 41 | Monakhali | 0 | 0 | 0.0003 | 0 | 0.7 | 0.047 | 0.007 | 6.667 | 6.667 |
| 42 | Monakhali | 0.075 | 0.005 | 0.0003 | 16.667 | 0.24 | 0.016 | 0.007 | 2.286 | 18.952 |
| 43 | Monakhali | 0.035 | 0.002 | 0.0003 | 7.778 | 0.57 | 0.038 | 0.007 | 5.429 | 13.206 |
| 44 | Monakhali | 0 | 0 | 0.0003 | 0 | 0.87 | 0.058 | 0.007 | 8.286 | 8.286 |
| 45 | Moahajanpur | 0 | 0 | 0.0003 | 0 | 0 | 0 | 0.007 | 0 | 0 |
| 46 | Moahajanpur | 0 | 0 | 0.0003 | 0 | 0.5 | 0.033 | 0.007 | 4.762 | 4.762 |
| 47 | Moahajanpur | 0.035 | 0.002 | 0.0003 | 7.778 | 1.89 | 0.126 | 0.007 | 18 | 25.778 |
| 48 | Moahajanpur | 0.075 | 0.005 | 0.0003 | 16.667 | 0.25 | 0.0167 | 0.007 | 2.381 | 19.048 |
| 49 | Moahajanpur | 0 | 0 | 0.0003 | 0 | 0.3 | 0.02 | 0.007 | 2.857 | 2.857 |
| 50 | Moahajanpur | 0.035 | 0.002 | 0.0003 | 7.778 | 0.53 | 0.035 | 0.007 | 5.048 | 12.825 |
| 51 | Moahajanpur | 0 | 0 | 0.0003 | 0 | 3.66 | 0.244 | 0.007 | 34.857 | 34.857 |
| 52 | Moahajanpur | 0 | 0 | 0.0003 | 0 | 0.17 | 0.011 | 0.007 | 1.619 | 1.619 |
| 53 | Moahajanpur | 0.035 | 0.002 | 0.0003 | 7.778 | 0.83 | 0.055 | 0.007 | 7.905 | 15.683 |
| 54 | Moahajanpur | 0.075 | 0.005 | 0.0003 | 16.667 | 0.5 | 0.033 | 0.007 | 4.762 | 21.429 |
| 55 | Moahajanpur | 0 | 0 | 0.0003 | 0 | 0.39 | 0.026 | 0.007 | 3.714 | 3.714 |
| 56 | Moahajanpur | 0 | 0 | 0.0003 | 0 | 0.5 | 0.033 | 0.007 | 4.762 | 4.762 |
| 57 | Moahajanpur | 0.035 | 0.002 | 0.0003 | 7.778 | 1.34 | 0.089 | 0.007 | 12.761 | 20.539 |
| 58 | Moahajanpur | 0 | 0 | 0.0003 | 0 | 1.27 | 0.084 | 0.007 | 12.095 | 12.095 |
| 59 | Moahajanpur | 0 | 0 | 0.0003 | 0 | 0.03 | 0.002 | 0.007 | 0.286 | 0.286 |
| 60 | Moahajanpur | 0.035 | 0.002 | 0.0003 | 7.778 | 2.15 | 0.143 | 0.007 | 20.476 | 28.254 |
| 61 | Moahajanpur | 0.035 | 0.002 | 0.0003 | 7.778 | 1.7 | 0.113 | 0.007 | 16.19 | 23.968 |
| 62 | Dariapur | 0 | 0 | 0.0003 | 0 | 0.97 | 0.065 | 0.007 | 9.238 | 9.238 |
| 63 | Dariapur | 0 | 0 | 0.0003 | 0 | 0 | 0 | 0.007 | 0 | 0 |
| 64 | Dariapur | 0 | 0 | 0.0003 | 0 | 0.23 | 0.015 | 0.007 | 2.19 | 2.19 |
| 65 | Dariapur | 0.035 | 0.002 | 0.0003 | 7.778 | 1.11 | 0.074 | 0.007 | 10.571 | 18.349 |
| 66 | Dariapur | 0 | 0 | 0.0003 | 0 | 2.25 | 0.15 | 0.007 | 21.429 | 21.428 |
| 67 | Dariapur | 0.175 | 0.012 | 0.0003 | 38.889 | 3.38 | 0.225 | 0.007 | 32.19 | 71.079 |
| 68 | Dariapur | 0.035 | 0.002 | 0.0003 | 7.778 | 0.45 | 0.03 | 0.007 | 4.286 | 12.063 |
| 69 | Dariapur | 0.175 | 0.012 | 0.0003 | 38.889 | 0 | 0 | 0.007 | 0 | 38.889 |
| 70 | Dariapur | 0 | 0 | 0.0003 | 0 | 1.36 | 0.091 | 0.007 | 12.952 | 12.952 |
| 71 | Dariapur | 0 | 0 | 0.0003 | 0 | 0.15 | 0.01 | 0.007 | 1.429 | 1.429 |
| 72 | Dariapur | 0.025 | 0.002 | 0.0003 | 5.556 | 0.07 | 0.005 | 0.007 | 0.667 | 6.222 |
| 73 | Dariapur | 0.035 | 0.002 | 0.0003 | 7.778 | 2.8 | 0.187 | 0.007 | 26.667 | 34.444 |
| 74 | Dariapur | 0.175 | 0.012 | 0.0003 | 38.889 | 3.1 | 0.207 | 0.007 | 29.524 | 68.412 |
| 75 | Dariapur | 0 | 0 | 0.0003 | 0 | 0.83 | 0.055 | 0.007 | 7.905 | 7.905 |

**Table S5:** Representation of the Categorical data and numeric values using Noncomparative Numerical Notation

| Sample Number | Latitude | X | Longitude | Y | Union | U |
| --- | --- | --- | --- | --- | --- | --- |
|  |  | (Lat-23) *10000 |  | (Long-88) *10000 |  |  |
| 1 | 23.6485 | 6485.02 | 88.596327 | 5963.27 | Bagoan | 0 |
| 2 | 23.63986 | 6398.62 | 88.612887 | 6128.87 | Bagoan | 0 |
| 3 | 23.63101 | 6310.13 | 88.609684 | 6096.84 | Bagoan | 0 |
| 4 | 23.62484 | 6248.35 | 88.606197 | 6061.97 | Bagoan | 0 |
| 5 | 23.63614 | 6361.44 | 88.611614 | 6116.14 | Bagoan | 0 |
| 6 | 23.63195 | 6319.46 | 88.612408 | 6124.08 | Bagoan | 0 |
| 7 | 23.63227 | 6322.72 | 88.612343 | 6123.43 | Bagoan | 0 |
| 8 | 23.65259 | 6525.88 | 88.587699 | 5876.99 | Bagoan | 0 |
| 9 | 23.64685 | 6468.47 | 88.592721 | 5927.21 | Bagoan | 0 |
| 10 | 23.66485 | 6648.51 | 88.604082 | 6040.82 | Bagoan | 0 |
| 11 | 23.64232 | 6423.15 | 88.581003 | 5810.03 | Bagoan | 0 |
| 12 | 23.64849 | 6484.86 | 88.583913 | 5839.13 | Bagoan | 0 |
| 13 | 23.66215 | 6621.49 | 88.581365 | 5813.65 | Bagoan | 0 |
| 14 | 23.66421 | 6642.09 | 88.603041 | 6030.41 | Bagoan | 0 |
| 15 | 23.65865 | 6586.47 | 88.613858 | 6138.58 | Bagoan | 0 |
| 16 | 23.66096 | 6609.62 | 88.616535 | 6165.35 | Bagoan | 0 |
| 17 | 23.64849 | 6484.9 | 88.650612 | 6506.12 | Bagoan | 0 |
| 18 | 23.62236 | 6223.62 | 88.626736 | 6267.36 | Bagoan | 0 |
| 19 | 23.61873 | 6187.3 | 88.624975 | 6249.75 | Bagoan | 0 |
| 20 | 23.6111 | 6110.96 | 88.623277 | 6232.77 | Bagoan | 0 |
| 21 | 23.62086 | 6208.61 | 88.606326 | 6063.26 | Bagoan | 0 |
| 22 | 23.65002 | 6500.22 | 88.647339 | 6473.39 | Bagoan | 0 |
| 23 | 23.64838 | 6483.79 | 88.643196 | 6431.96 | Bagoan | 0 |
| 24 | 23.62101 | 6210.14 | 88.617398 | 6173.98 | Bagoan | 0 |
| 25 | 23.62306 | 6230.57 | 88.622459 | 6224.59 | Bagoan | 0 |
| 26 | 23.61501 | 6150.09 | 88.622601 | 6226.01 | Bagoan | 0 |
| 27 | 23.64359 | 6435.9 | 88.587183 | 5871.83 | Bagoan | 0 |
| 28 | 23.6239 | 6239.01 | 88.616447 | 6164.47 | Bagoan | 0 |
| 29 | 23.6548 | 6548 | 88.590563 | 5905.63 | Bagoan | 0 |
| 30 | 23.67567 | 6756.71 | 88.608209 | 6082.09 | Monakhali | 1 |
| 31 | 23.675 | 6749.96 | 88.613496 | 6134.96 | Monakhali | 1 |
| 32 | 23.67068 | 6706.79 | 88.603599 | 6035.99 | Monakhali | 1 |
| 33 | 23.67327 | 6732.72 | 88.615454 | 6154.54 | Monakhali | 1 |
| 34 | 23.6683 | 6682.98 | 88.618288 | 6182.88 | Monakhali | 1 |
| 35 | 23.67703 | 6770.33 | 88.615477 | 6154.77 | Monakhali | 1 |
| 36 | 23.71001 | 7100.09 | 88.613214 | 6132.14 | Monakhali | 1 |
| 37 | 23.71361 | 7136.13 | 88.613431 | 6134.31 | Monakhali | 1 |
| 38 | 23.67663 | 6766.32 | 88.604662 | 6046.62 | Monakhali | 1 |
| 39 | 23.65229 | 6522.86 | 88.636257 | 6362.57 | Monakhali | 1 |
| 40 | 23.68943 | 6894.3 | 88.676368 | 6763.68 | Monakhali | 1 |
| 41 | 23.67403 | 6740.27 | 88.621861 | 6218.61 | Monakhali | 1 |
| 42 | 23.67053 | 6705.31 | 88.610899 | 6108.99 | Monakhali | 1 |
| 43 | 23.67447 | 6744.68 | 88.610654 | 6106.54 | Monakhali | 1 |
| 44 | 23.67781 | 6778.11 | 88.612818 | 6128.18 | Monakhali | 1 |
| 45 | 23.66043 | 6604.3 | 88.652199 | 6521.99 | Moahajanpur | 2 |
| 46 | 23.67664 | 6766.37 | 88.667654 | 6676.54 | Moahajanpur | 2 |
| 47 | 23.68344 | 6834.35 | 88.674885 | 6748.85 | Moahajanpur | 2 |
| 48 | 23.68101 | 6810.08 | 88.677897 | 6778.97 | Moahajanpur | 2 |
| 49 | 23.6526 | 6526.02 | 88.66817 | 6681.7 | Moahajanpur | 2 |
| 50 | 23.65397 | 6539.69 | 88.673069 | 6730.69 | Moahajanpur | 2 |
| 51 | 23.65045 | 6504.47 | 88.672408 | 6724.08 | Moahajanpur | 2 |
| 52 | 23.65803 | 6580.3 | 88.677425 | 6774.25 | Moahajanpur | 2 |
| 53 | 23.68009 | 6800.91 | 88.68304 | 6830.4 | Moahajanpur | 2 |
| 54 | 23.7 | 7000.03 | 88.6819 | 6819 | Moahajanpur | 2 |
| 55 | 23.69616 | 6961.63 | 88.675269 | 6752.69 | Moahajanpur | 2 |
| 56 | 23.69247 | 6924.67 | 88.674179 | 6741.79 | Moahajanpur | 2 |
| 57 | 23.65285 | 6528.5 | 88.677082 | 6770.82 | Moahajanpur | 2 |
| 58 | 23.6556 | 6555.99 | 88.675297 | 6752.97 | Moahajanpur | 2 |
| 59 | 23.65316 | 6531.59 | 88.677802 | 6778.02 | Moahajanpur | 2 |
| 60 | 23.687 | 6870.03 | 88.676586 | 6765.86 | Moahajanpur | 2 |
| 61 | 23.68524 | 6852.43 | 88.674347 | 6743.47 | Moahajanpur | 2 |
| 62 | 23.68105 | 6810.52 | 88.610066 | 6100.66 | Dariapur | 3 |
| 63 | 23.69166 | 6916.63 | 88.609843 | 6098.43 | Dariapur | 3 |
| 64 | 23.70057 | 7005.68 | 88.604183 | 6041.83 | Dariapur | 3 |
| 65 | 23.70387 | 7038.73 | 88.600354 | 6003.54 | Dariapur | 3 |
| 66 | 23.70938 | 7093.78 | 88.609769 | 6097.69 | Dariapur | 3 |
| 67 | 23.70933 | 7093.31 | 88.607583 | 6075.83 | Dariapur | 3 |
| 68 | 23.70175 | 7017.5 | 88.5996 | 5996 | Dariapur | 3 |
| 69 | 23.70792 | 7079.21 | 88.609099 | 6090.99 | Dariapur | 3 |
| 70 | 23.70618 | 7061.8 | 88.594986 | 5949.86 | Dariapur | 3 |
| 71 | 23.71023 | 7102.26 | 88.596147 | 5961.47 | Dariapur | 3 |
| 72 | 23.71138 | 7113.78 | 88.610055 | 6100.55 | Dariapur | 3 |
| 73 | 23.70967 | 7096.68 | 88.610884 | 6108.84 | Dariapur | 3 |
| 74 | 23.70961 | 7096.05 | 88.605356 | 6053.56 | Dariapur | 3 |
| 75 | 23.71138 | 7113.77 | 88.607441 | 6074.41 | Dariapur | 3 |

**Table S6**: Safe water consumption limit (L/day) for individual sampling points

| **Sample No.** | **Union** | **Con. (As) (mg/l)** | **Con. (Fe)(mg/l)** | **Safe consumption (L/day)** |
| --- | --- | --- | --- | --- |
| 1 | Baguan | 0.075 | 0.18 | 0.14 |
| 2 | Baguan | 0 | 0.46 | 0.54 |
| 3 | Baguan | 0.075 | 1.56 | 0.14 |
| 4 | Baguan | 0 | 0.47 | 0.53 |
| 5 | Baguan | 0 | 1 | 0.25 |
| 6 | Baguan | 0.175 | 0.51 | 0.06 |
| 7 | Baguan | 0 | 1.35 | 0.18 |
| 8 | Baguan | 0 | 0.14 | 1.77 |
| 9 | Baguan | 0 | 0.88 | 0.28 |
| 10 | Baguan | 0.075 | 1.99 | 0.12 |
| 11 | Baguan | 0.175 | 0.59 | 0.06 |
| 12 | Baguan | 0 | 0.13 | 1.91 |
| 13 | Baguan | 0 | 0.2 | 1.24 |
| 14 | Baguan | 0.075 | 0 | 0.14 |
| 15 | Baguan | 0 | 0.59 | 0.42 |
| 16 | Baguan | 0.075 | 0.78 | 0.14 |
| 17 | Baguan | 0 | 0.35 | 0.71 |
| 18 | Baguan | 0 | 0.81 | 0.31 |
| 19 | Baguan | 0 | 0.66 | 0.38 |
| 20 | Baguan | 0.175 | 2.23 | 0.06 |
| 21 | Baguan | 0.075 | 0.17 | 0.14 |
| 22 | Baguan | 0 | 0.09 | 2.76 |
| 23 | Baguan | 0.175 | 0.15 | 0.06 |
| 24 | Baguan | 0 | 0.74 | 0.34 |
| 25 | Baguan | 0.035 | 1.21 | 0.21 |
| 26 | Baguan | 0.075 | 2.5 | 0.10 |
| 27 | Baguan | 0 | 0.06 | 4.14 |
| 28 | Baguan | 0.035 | 2.4 | 0.10 |
| 29 | Baguan | 0.075 | 2.76 | 0.09 |
| 30 | Monakhali | 0 | 2.72 | 0.09 |
| 31 | Monakhali | 0.075 | 0.8 | 0.14 |
| 32 | Monakhali | 0 | 0.36 | 0.69 |
| 33 | Monakhali | 0.035 | 1.32 | 0.19 |
| 34 | Monakhali | 0 | 0.23 | 1.08 |
| 35 | Monakhali | 0.035 | 0.28 | 0.30 |
| 36 | Monakhali | 0.035 | 2.39 | 0.10 |
| 37 | Monakhali | 0 | 0.26 | 0.95 |
| 38 | Monakhali | 0 | 0.23 | 1.08 |
| 39 | Monakhali | 0.075 | 0.69 | 0.14 |
| 40 | Monakhali | 0 | 1.87 | 0.13 |
| 41 | Monakhali | 0 | 0.7 | 0.35 |
| 42 | Monakhali | 0.075 | 0.24 | 0.14 |
| 43 | Monakhali | 0.035 | 0.57 | 0.30 |
| 44 | Monakhali | 0 | 0.87 | 0.29 |
| 45 | Moahajanpur | 0 | 0 | 8.27 |
| 46 | Moahajanpur | 0 | 0.5 | 0.50 |
| 47 | Moahajanpur | 0.035 | 1.89 | 0.13 |
| 48 | Moahajanpur | 0.075 | 0.25 | 0.14 |
| 49 | Moahajanpur | 0 | 0.3 | 0.83 |
| 50 | Moahajanpur | 0.035 | 0.53 | 0.30 |
| 51 | Moahajanpur | 0 | 3.66 | 0.07 |
| 52 | Moahajanpur | 0 | 0.17 | 1.46 |
| 53 | Moahajanpur | 0.035 | 0.83 | 0.30 |
| 54 | Moahajanpur | 0.075 | 0.5 | 0.14 |
| 55 | Moahajanpur | 0 | 0.39 | 0.64 |
| 56 | Moahajanpur | 0 | 0.5 | 0.50 |
| 57 | Moahajanpur | 0.035 | 1.34 | 0.19 |
| 58 | Moahajanpur | 0 | 1.27 | 0.20 |
| 59 | Moahajanpur | 0 | 0.03 | 8.27 |
| 60 | Moahajanpur | 0.035 | 2.15 | 0.12 |
| 61 | Moahajanpur | 0.035 | 1.7 | 0.15 |
| 62 | Dariapur | 0 | 0.97 | 0.26 |
| 63 | Dariapur | 0 | 0 | 8.27 |
| 64 | Dariapur | 0 | 0.23 | 1.08 |
| 65 | Dariapur | 0.035 | 1.11 | 0.22 |
| 66 | Dariapur | 0 | 2.25 | 0.11 |
| 67 | Dariapur | 0.175 | 3.38 | 0.06 |
| 68 | Dariapur | 0.035 | 0.45 | 0.30 |
| 69 | Dariapur | 0.175 | 0 | 0.06 |
| 70 | Dariapur | 0 | 1.36 | 0.18 |
| 71 | Dariapur | 0 | 0.15 | 1.65 |
| 72 | Dariapur | 0.025 | 0.07 | 0.43 |
| 73 | Dariapur | 0.035 | 2.8 | 0.09 |
| 74 | Dariapur | 0.175 | 3.1 | 0.06 |
| 75 | Dariapur | 0 | 0.83 | 0.30 |


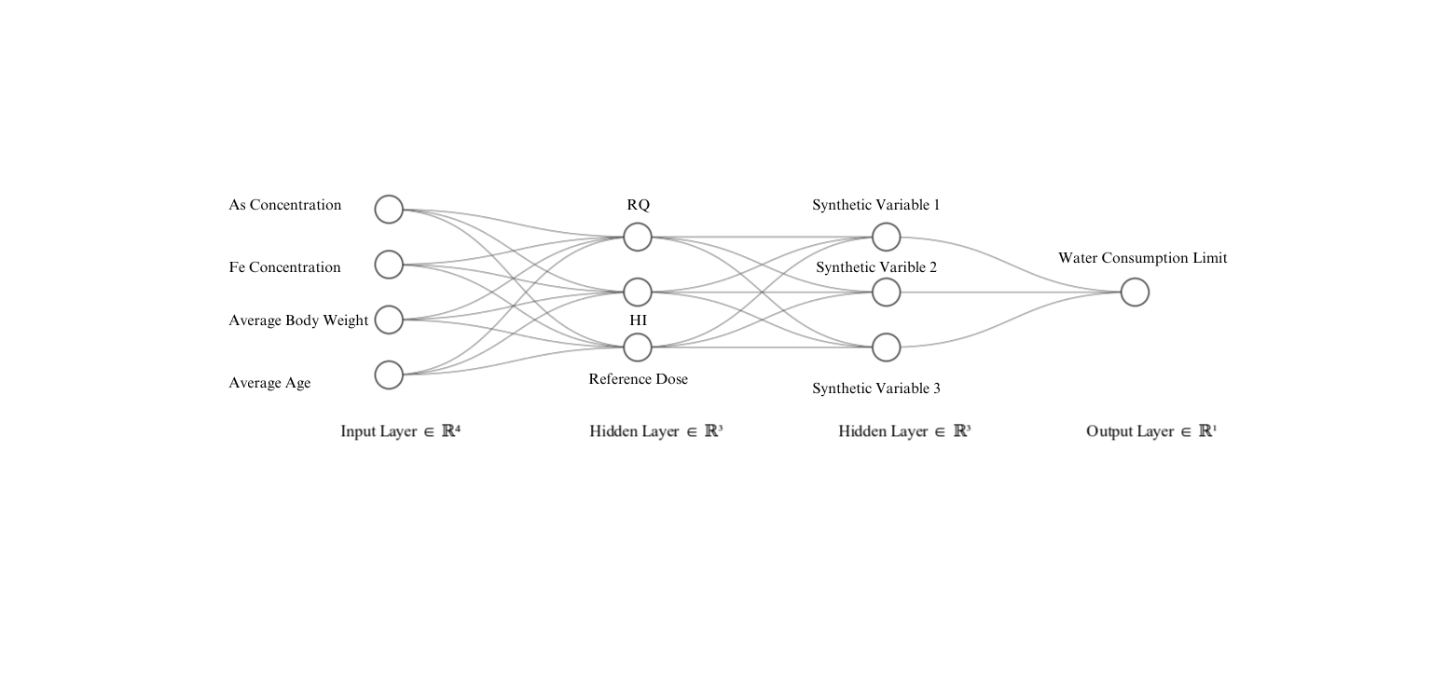


**Figure S1** Flow-diagram of Artificial Neural Network (ANN) processes and data clustering using the driven data of groundwater of the study area along with the consideration of THQ values, defining the internal and hidden nature of the data.

1. Sample size = 90%


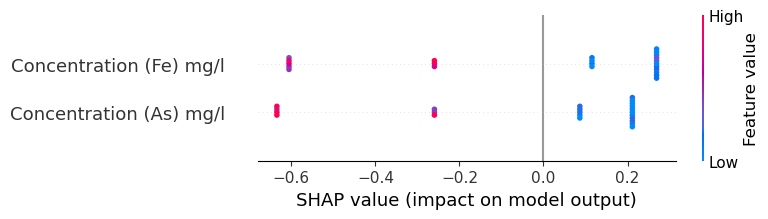


1. Sample size=10%


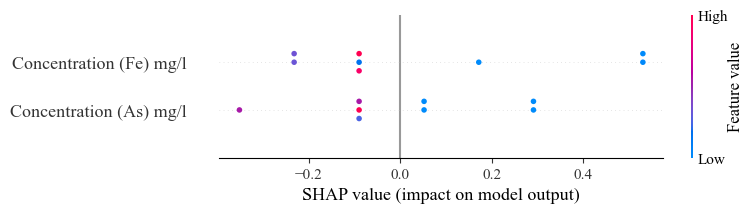


**Figure S2** The SHAP values and influence over the output **(a)** Sample size = 90% (left); **(b)** Sample size=10% (right). SHAP values exert a positive influence on predictions when they are positive values and a negative influence when they are negative values. The magnitude of these values serves as an indicator of the strength of their impact.
